# miR615‐3p inhibited FBLN1 and osteogenic differentiation of umbilical cord mesenchymal stem cells by associated with YTHDF2 in a m^6^A‐miRNA interaction manner

**DOI:** 10.1111/cpr.13607

**Published:** 2024-02-14

**Authors:** Haoqing Yang, Wanqing Wang, Huina Liu, Chen Zhang, Yangyang Cao, Lujue Long, Xiao Han, Yuejun Wang, Fei Yan, Guoqing Li, Mengyuan Zhu, Luyuan Jin, Zhipeng Fan

**Affiliations:** ^1^ Laboratory of Molecular Signaling and Stem Cells Therapy, Beijing Key Laboratory of Tooth Regeneration and Function Reconstruction Capital Medical University School of Stomatology Beijing China; ^2^ Jiangsu Province Key Laboratory of Oral Diseases Nanjing Medical University Nanjing China; ^3^ Department of General Dentistry and Integrated Emergency Dental Care, Beijing Stomatological Hospital Capital Medical University Beijing China; ^4^ Xiangya Stomatological Hospital and School of Stomatology Central South University Changsha China; ^5^ Beijing Laboratory of Oral Health Capital Medical University Beijing China; ^6^ Research Unit of Tooth Development and Regeneration Chinese Academy of Medical Sciences Beijing China

## Abstract

To investigate the role and mechanism of FBLN1 in the osteogenic differentiation and bone regeneration by using umbilical cord mesenchymal stem cells (WJCMSCs). We found that FBLN1 promoted osteogenic differentiation of WJCMSCs and WJCMSC‐mediated bone regeneration. It was showed that there was an m^6^A methylation site in 3′UTR of FBLN1 mRNA, and the mutation of the m^6^A site enhanced the stability of FBLN1 mRNA, subsequently fostering the FBLN1 enhanced osteogenic differentiation of WJCMSCs. YTHDF2 was identified as capable of recognizing and binding to the m^6^A site, consequently inducing FBLN1 instability and repressed the osteogenic differentiation of WJCMSCs. Meanwhile, miR‐615‐3p negatively regulated FBLN1 by binding FBLN1 3′UTR and inhibited the osteogenic differentiation of WJCMSCs and WJCMSC‐mediated bone regeneration. Then, we discovered miR‐615‐3p was found to regulate the functions of FBLN1 facilitated by YTHDF2 through an m^6^A‐miRNA regulation mechanism. We demonstrated that FBLN1 is critical for regulating the osteogenic differentiation potentials of WJCMSCs and have identified that miR615‐3p mediated the decay of FBLN1 mRNA which facilitated by m^6^A reading protein YTHDF2. This provided a novel m^6^A‐miRNA epigenetic regulatory pattern for MSC regulation and bone regeneration.

## Graphical abstract

FBLN1 plays an important role during osteogenic differentiation and bone regeneration of MSCs. Both the m^6^A modification site and the miR615‐3p‐binding site exist on the 3′UTR of FBLN1. miR‐615‐3p can target the degradation of FBLN1 mRNA. The m^6^A modification site on the FBLN1 3′UTR can be recognized and degraded by YTHDF2. miR‐615‐3p, FBLN1 3′UTR, and YTHDF2 can form a complex. The regulatory effect of miR‐615‐3p on FBLN1 depends on the combination of YTHDF2 and FBLN1 3′UTR.

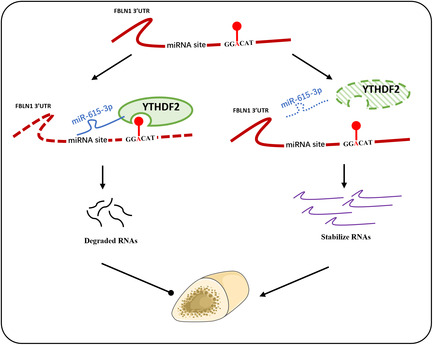

## 1 INTRODUCTION

Bone defects present a significant clinical challenge, requiring functional reconstruction and aesthetic remodelling. While autografts remain the preferred tissue replacement material, they are limited by the need for additional surgeries, donor site morbidity, and restricted availability. Bone tissue engineering strategies aim to facilitate the regeneration of bone tissue that has been compromised. With the advancement of tissue engineering technology, the utilization of mesenchymal stem cells (MSCs) for bone tissue regeneration has emerged as a promising technique.^1^ MSCs have gained prominence as the most extensively employed stem cell type in therapeutic applications, primarily due to their ethically sourced cells, ease of isolation and expansion in vitro, as well as their minimal immunogenicity. Notably, WJCMSCs offer distinctive advantages, as they can be sourced from medical waste, thereby obviating the need for painful invasive procedures. Furthermore, WJCMSCs possess superior proliferation, differentiation, and transfection abilities compared to alternative MSCs.^2^, ^3^

The extracellular matrix (ECM) environment provides MSCs with a vital niche for their survival, which playing a pivotal role in facilitating the functionality of MSCs.^4^ Identifying matrix proteins that enhance the formation and stability of ECM complexes and understanding their roles in bone regeneration is crucial. The Fibulin (FBLN) family of extracellular matrix proteins assists in the assembly and stabilization of ECM complexes, significantly impacting elastic fibre and basement membrane formation.^5^, ^6^, ^7^ The protein family has seven members, FBLN1‐7. These members have some structural similarities. ‘Domain III’ or ‘FC domain’: A spherical domain present at the carboxyl terminal, which is a common feature of the FBLN family.^7^, ^8^, ^9^ ‘Domain II’: Variable number of calcium‐binding epithelial growth factor modules. ‘Domain I’: Variable region at the end of the amino group. FBLN s are classified into two subgroups based on the overall protein structure.^10^ The first subgroup includes FBLN‐1 and ‐2, which have three anaphylatoxins (AT) modules within their first domain. The second subgroup comprises five members of FBLN 3–7, with the first three members referred to as ‘short Fibulins’ due to their molecular weights falling within the range of 50 and 60 kDa.^11^, ^12^, ^13^ Functionally, FBLNs are important in the occurrence and development of bone.

Unlike FBLN2‐7, FBLN1 is a critical protein for bone development, widely recognized for its deposition in the extracellular matrix (ECM) and close association with osteoblasts, playing a significant role in bone formation and regeneration.^14^, ^15^ Mutant mouse embryos lacking FBLN1 have abnormal skeletal development and reduced bone mass.^16^ And it has been reported to improve the osteogenic signalling activity of Bmp‐2 during osteogenesis, impacting the expression of the essential osteogenic transcription factor OSX. FBLNs expression pattern is similar to DMP1, suggesting that FBLN1 is crucial in cell‐matrix and cell‐to‐cell interactions during dentin mineralization.^9^, ^17^ Given its critical role in mediating cellular interactions and signalling pathways essential for osteoblast function and skeletal development, elucidating the precise mechanisms of FBLN1 in MSCs can lead to breakthroughs in bone tissue engineering and regenerative medicine.

In the post‐transcriptional regulation of eukaryotic mRNA, 3′UTR plays an important role. It is involved in regulating mRNA stability and degradation rate, controlling mRNA utilization efficiency, and determining the site and efficiency of mRNA translation. Current studies show many dynamic and reversible chemical modifications on RNA, which is important for posttranscriptional gene regulation in ECM proteins.^4^ N6‐methyladenosine (m^6^A) represents the most prevalent internal mRNA modification, constituting the largest internally modified base in mammalian systems. It plays a significant role in posttranscriptional gene regulation, particularly in the context of ECM proteins.^4^, ^18^ Recent studies showed that m^6^A RNA modification is involved in the osteogenic differentiation of MSCs.^19^, ^20^ However, it is unknown whether the m^6^A alteration affects the expression of FBLN1 and the osteogenic differentiation of MSCs. m^6^A modifications on mRNA are post‐transcriptionally introduced, removed, and recognized by three key types of proteins: m^6^A methyltransferases, often referred to as ‘writers’, which install the modification; m^6^A demethylases, known as ‘erasers’, which remove it; and m^6^A‐specific binding proteins, termed ‘readers’, which recognize and interact with the modified RNA. The YTH‐domain‐containing family is regarded as the main ‘Reader’ proteins of RNA m^6^A modification and is mediated tissue regeneration, such as self‐renewal^21^ and bone metabolism^22^, ^23^ in a m^6^A dependent manner. As a key ‘Reader’ of RNA m^6^A modification, the regulatory effect and mechanism of YTHDF2 on MSCs are still unclear.

Previous literature suggested that the 3′UTR region of mRNA is the target binding region of miRNA, and the overall distribution of m^6^A peaks and miRNA‐binding sites in 3′UTR are negatively correlated, and m^6^A needs a certain spatial separation to affect the function of downstream binding miRNAs.^24^ In particular, the proportion of highly expressed miRNAs in the target transcripts containing m^6^A is much higher. These data indicate that the level of miRNAs may control the methylation of their target gene transcripts; that is, m^6^A modification in the target gene mRNA may be involved in the functional regulation of miRNAs.^24^, ^25^, ^26^

In this study, we explored the role and underlying mechanism of FBLN1 in influencing the osteogenic differentiation capability by using WJCMSCs. We discovered that FBLN1 promoted osteogenic differentiation of WJCMSCs and WJCMSC‐mediated bone regeneration. Mechanistically, we found that the 3′UTR of FBLN1 contains m^6^A sites and miRNA‐binding sites. Then YTHDF2 interplays with miR615‐3p to regulate FBLN1 and therefore plays critical function in osteogenic differentiation ability of WJCMSCs.

## 2 MATERIALS AND METHODS

### 2.1 Ethics statement

All animal experiments were conducted in accordance with the approved regulations of the Beijing Stomatological Hospital, Capital Medical University (Ethical Committee Agreement, Beijing Stomatological Hospital Ethics Review No. KQYY‐2022‐001). The animals were housed in adjacent identical enclosures and provided continuous access to a standard commercial diet and drinking water. All investigations involving human mesenchymal stem cells adhered to the ISSCR ‘Guidelines for the Conduct of Human Embryonic Stem Cell Research’. This study adhered to the ARRIVE guidelines for preclinical animal studies.

### 2.2 Cell culture and osteogenic differentiation

Human WJCMSCs were purchased from Cyagen Biosciences (Guang zhou, People's Republic of China). Human embryonic kidney 293 T were obtained from ATCC (CRL‐3216, USA). Cells were incubated in complete DMEM supplemented with 10% FBS (10099141C, Invitrogen, USA), and penicillin–streptomycin‐glutamine mix (10378016, Invitrogen, USA), under 5% CO_2_ at 37°C. The culture medium was refreshed every three days. For osteoblasts induction, cells (2.0×10^5^ cells/well) were seeded into 6‐well plates and allowed to grow to 80% confluence. Then cells were grown with the StemPro® Osteogenesis Differentiation medium (A1007201, Invitrogen, USA) for mineralization induction. The ALP activity assay and Alizarin red detection were performed as previously described.^27^

### 2.3 Plasmid construction and viral infection

The plasmids were synthesized using conventional methods, and the accuracy of each construct was verified by gene sequencing. Human FBLN1 cDNA with HA tag was synthesized and subcloned into the pQCXIN (Clontech, USA) lentiviral vector to produce an overexpression vector. The cDNA of human YTHDF2 with HA tag, FBLN1 with CDS and 3′UTR (WT‐FBLN1), and FBLN1 cDNA with CDS, 3′UTR and the deletion of m^6^A sites (Mut‐FBLN1) were subcloned into the pCDH lentiviral vector (Clontech) separately. We designed sequences with mutations that delete m^6^A sites (HA‐Mut1‐FBLN1), mutants that delete miR615‐3p‐binding sites (HA‐Mut2‐FBLN1), and mutants that delete both m^6^A sites and miR615‐3p‐binding sites (HA‐Mut3‐FBLN1). These sequences were subcloned into the pCDH lentiviral vector (Clontech) independently.

Short hairpin RNAs (shRNA) lentiviral vector targeting FBLN1, YTHDF2, miR615‐3p mimic, or miR615‐3p inhibitor and the control lentiviral vector were generated by GenePharma. WJCMSCs were subjected to lentiviral infection for a duration of 12 h in the presence of polybrene (BL628A, Biosharp, People's Republic of China). 48 h after infection, the infected WJCMSCs were subjected to antibiotic selection. The target sequences for the shRNAs were subsequently enumerated as follows:

FBLN1 shRNA (FBLN1sh), 5′‐GCTGCCGACCCAAGCTACAGT‐3′;

YTHDF2 shRNA (YTHDF2sh), 5′‐ GCGGGTCCATTACTAGTAACA‐3′;

Control shRNA (Consh), 5′‐TTCTCCGAACGTGTCACGTTTC‐3′;

hsa‐miR‐615‐3p inhibitor, 5′‐AAGAGGGAGACCCAGGCTCGGA‐3′;

hsa‐miR‐615‐3p mimic, 5′‐TCCGAGCCTGGGTCTCCCTCTT‐3′;

### 2.4 Western blot analysis

Proteins were extracted with lysis containing protease inhibitors (P1265, Applygen, People's Republic of China), phosphatase inhibitors (P1260, Applygen), RNase A (R301, Vazyme, People's Republic of China), and RNase inhibitor (RL301, Vazyme). Western blot assays were performed as previously described.^27^ The primary antibodies used in this study include YTHDF2 (Proteintech, USA); HA tag (51064‐2‐AP, Proteintech); AGO2 (Proteintech); YTHDF3 (25537‐1‐AP, Proteintech); FBLN1(Abcam); DSPP (Bioss); DMP1 (Bioss); GAPDH (C1312, Applygen); and β‐actin (C1312, Applygen).

### 2.5 Co‐immunoprecipitation (Co‐IP)

Samples were extracted from 10 cm dish per sample using IP lysis buffer (87,788, Invitrogen, USA) containing protease inhibitor cocktail (Applygen). The cell lysates were incubated with specific primary antibodies or rabbit isotype control IgG with agitated rotation at 4°C overnight. Then protein A/G magnetic beads were added to the samples, and they were incubated for 2 h. The beads were washed with lysis buffer three times to remove unbound proteins. Then western blotting analysis using corresponding antibodies was applied.

### 2.6 Real‐time reverse transcriptase‐PCR

Total RNA isolation (R711‐01, Vazyme) and cDNA synthesis (HiScript III RT SuperMix for qPCR (+gDNA wiper)), (R323‐01, Vazyme) were conducted according to the manufacture's protocol (Vazyme). Real‐time PCR was done using MagicSYBR Mixture (CW3008, CWBIO, People's Republic of China). Table S1 summarizes the primers used in this study. For miRNA detection, the reverse transcription–polymerase chain reaction (RT‐PCR) was performed using TaqMan MicroRNA Reverse Transcription Kit (4366596, Invitrogen, USA). The TaqMan probes of hsa‐miR‐615 (Assay ID: 001960, Applied Biosystems, USA) were used for the detection of specific microRNAs, and U6 (Assay ID: 001973, Applied Biosystems) was used as an loading control. The steady‐state level of mature miRNAs was determined using miR‐specific TaqMan™ MicroRNA Assay Kits (Applied Biosystems). Quantitative real‐time PCR (qPCR) was performed with a BioRad CFX96 Real Time PCR Detection System (BioRad, USA). Each reaction was conducted in triplicate, and the entire experimental procedure was replicated three times to ensure the reliability and consistency of the results. Relative expression was calculated by using the 2^−ΔΔCt^ method.

### 2.7 Cell Immunofluorescence staining

For cell immunofluorescence staining, WJCMSCs were processed according to the manufacture's protocol (Abcam, USA). Then the processed MSCs were incubated with primary antibodies of FBLN1(ab211536, Abcam, USA), YTHDF2 (24744‐1‐AP, Proteintech), and AGO2 (67934‐1‐Ig, Proteintech), respectively, overnight at 4°C. Next, the MSCs were stained with fluorescent secondary antibodies (Alexa Fluor, Invitrogen, USA) for 2 h. DAPI (D1306, Invitrogen) was used for nucleus staining. Fluorescence pictures were captured through a fluorescence microscope or a confocal microscope.

### 2.8 Creation of critical‐sized calvarial defect, WJCMSCs sheet preparation, and implantation

WJCMSCs sheet preparation: MSCs were seeded into six‐well plates at a cell density of 2 × 10^5^/well. Standard medium (50 mg/mL L‐ascarbicacid2‐phosphate, 10 mmol/L‐glycerophosphate and 100 nmol/L dexamethasone) (Sigma‐Aldrich, USA) was added into the culture medium for 2 weeks. After that, the MSCs sheet was formed. Use a scraper to scrape the MSCs sheet for backup use. Nude rats (male, 4 months old) were used in these animal experiments. Anaesthesia was induced with intraperitoneal injection of ketamine/xylazine. According to previous reports, we prepared a critical‐sized defect in the calvaria.^28^ In short, a sagittal incision was made at the calvarium of the nude rats, and the skin and periosteum layers were dissected. When the parietal bones were fully exposed, Critical‐sized defects (5 mm in diameter) were made on both sides of the middle cranial suture. Subsequently, four groups of MSCs sheet were placed into the defect site: (1) WJCMSCs/Vector group; (2) WJCMSCs/FBLN1 group; and (3) WJCMSCs/Consh group; (4) WJCMSCs/miR‐615‐3p‐inhibitor group. The periosteum and skin were overlaid and firmly sutured with absorbable line (4/0). 12 weeks after transplantation, the transplanted tissues were harvested.

### 2.9 Histological and immunofluorescence analyses

The slices were selected randomly and then generating a randomization sequence using a simple random sampling method. To obtain the credible data, we minimized the potential confounding factors between groups. The specimens were fixed in 10% formalin. Then the fixed specimens were embedded in paraffin, and the thickness of the sections was 5 𝜇m. Hematoxylin and eosin (H&E) was used for structural analyses. Immunofluorescence assay was used to evaluate DSPP, DMP1, and OCN expression. In brief, the sections underwent antigen retrieval as previously described and were subsequently incubated with DSPP (bs‐10316R, Bioss, People's Republic of China), DMP1 (bs‐12359R, Bioss), and OCN (bs‐0470R, Bioss). Next, the sections were subjected to staining using fluorescent secondary antibodies (Alexa Fluor, Invitrogen, USA) for 2 h. DAPI (D1306, Invitrogen) was used for nucleus staining. Fluorescence pictures were captured through a fluorescence microscope or a confocal microscope.

### 2.10 Micro‐computed tomography (micro‐CT) scanning evaluation

In situ bone regeneration (critical size calvarial defect in nude rats) was evaluated by Micro‐CT scan (Siemens, Berlin, Germany) (an operating voltage of 50 kVp and a pitch of 0.14 mm were set for the CT machine). By fitting the 3D reconstructed images using Geomagic 12, the growth volume of newly regenerated tissue can be obtained and calculated. The results were normalized by using the smallest volume from the control group. Student's *t*‐test was used to analyse the data. The samples were selected randomly and then generating a randomization sequence using a simple random sampling method. To obtain the credible data, we minimized the potential confounding factors between groups.

### 2.11 Dual‐luciferase assay

Luciferase reporters were obtained by cloning WT FBLN1 3′UTR or Mut FBLN1 3′UTR into pmirGLO Dual‐Luciferase miRNA Target Expression Vector (Promega, WI, USA). miR‐615‐3p mimics, mimic control, miR‐615‐3p inhibitor, and a negative control of scramble RNA are purchased from GenePharma (GenePharma). 293 T cells were co‐transfected with the pmirGLO plasmids, and miRNA mimics or miRNA inhibitor, using the lipofectamine 3000 reagent (L3000001, Invitrogen, USA). The luminescence as measured using the Dual‐Glo® Luciferase Assay System 48 h after transfection (E2940, Promega, USA).

### 2.12 Biotin‐RNA pull‐down

To confirm the binding of miR‐615‐3p to the FBLN1 3′UTR, biotin‐RNA pull down and RT‐qPCR were used. Briefly, biotin‐labelled miR‐615‐3p was transfected into WJCMSCs and 293 T cells using lipofectamine 3000 (Invitrogen). At 36 h post‐transfection, cell lysis was performed using IP lysis buffer (Invitrogen), and immunoprecipitation was carried out using MyOne streptavidin beads (65601, Invitrogen). The bound RNA was then extracted, reverse transcribed, and qPCR was used to detect the presence of FBLN1 3′UTR. To identify the miR‐615‐3p‐interacting proteins in WJCMSCs, biotin‐RNA pull down and iTRAQ assays (Isobaric Tag for Relative Absolute Quantitation) were performed. Briefly, the RNA‐binding proteins were sequenced according to the manufacturer's instructions for the isobaric tag for relative and absolute quantitation (iTRAQ) kit (AB SCIEX, CA, USA) and identified by AB Sciex Protein Pilot 4.0 (AB Sciex, Concord, Ontario, Canada), and protein identification was determined by the UniProt database.

### 2.13 RNA stability assay

RNA stability was evaluated by Actinomycin D assays. Briefly, cells at 80% confluence were exposed to 2 μg/mL actinomycin D at time points of 0, 3, and 6 h. RNA totals were extracted, and qPCR assays were performed as described previously. The 2^−ΔΔCT^ method was used to calculate the relative RNA expression levels.

### 2.14 RNA immunoprecipitation assays (RIP‐qPCR)

Cells were lysed using RIP buffer. After centrifugation, the supernatant was harvested. 5% of the total volume was set aside as Input sample. The remaining supernatant was subjected to RNA immunoprecipitation with YTHDF2 (Proteintech) or IgG (bs‐0295P, Bioss) antibodies and recorded as the beads samples, respectively. Magnetic beads (HY‐K0202, MCE, USA) were incubated with the beads samples. The RNA from the beads and Input samples were washed and extracted. Then bound RNAs were extracted, purified, and subjected to RT‐qPCR assay. Primers for RIP‐qPCR are listed in Table S1.

### 2.15 MeRIP‐Seq and MeRIP‐qPCR

MeRIP‐seq were applied to map the m^6^A‐methylated RNA. Total RNA of WJCMSCs was extracted using Trizol (15,596,026, Invitrogen, USA). Adenylated RNA (polyA+RNA) was enriched from total RNA using the GenElute mRNA miniprep kit (MRN10, Sigma‐Aldrich, USA). The adenylated RNA was then fragmented and precipitated, followed by resuspension in MeRIP buffer. The m^6^A‐methylated RNA was immunoprecipitated using m^6^A‐specific antibody (A19841, abclonal, People's Republic of China). Approximately 2.5 μg of fragmented total RNA was retained as Input samples. The IP and Input group RNA from WJCMSCs was sequenced on a Illumina sequencing system (Illumina Inc., USA) using a genome sequencing kit. The raw reads of MeRIP‐seq datasets were mapped to human (hg19) reference genome using HISAT2 software. The exomePeak (Version 3.8) software was applied for peak calling. Bedtools (Version 2.25.0) was used to annotate the m^6^A peaks. The sequence of motifs enriched in m^6^A peak regions was identified using Homer (version 4.10). The MeRIP enriched regions (peaks) were visualised by the Integrative Genomics Viewer (IGV, Broad Institute, MA, USA).

For MeRIP‐qPCR, RNA from the IP and input groups of WJCMSCs were processed as described previously. The enrichment of m^6^A was measured with quantitative RT‐PCR. Primers for m^6^A‐RIP‐qPCR are listed in Table S1.

### 2.16 CLIP‐qPCR

The four‐thiouridine (C3722, APExBIO, USA) was added to the cell culture medium 16 h before UV irradiation. The cells were irradiated under a UV cross‐linker (Spectrolinke XL‐1000 UV crosslinker, Thomas Scientific, USA) at a dose of 0.15 J/cm^2^ for 10 min at 4°C. Cells were then lysed in RIPA buffer, and 1% volume of the cell lysates were retained as Input. The remaining cell lysates were immunoprecipitated with antibodies against m^6^A (abclonal) or IgG (Bioss) overnight at 4°C. Immunoprecipitation complexes were collected with magnetic beads (MCE). The magnetic beads were immobilized, followed by elution of the immunoprecipitant containing the specific protein along with its bound RNA. Subsequently, the remaining RNA was extracted for qRT‐PCR analysis using previously established protocols. Establish and run PCR reactions using ChamQ SYBR Colour qPCR Master Mix (Q411‐02, Vazyme, People's Republic of China). Normalize the enrichment values to the input sample by 2^−ΔΔCT^ method calculation.

### 2.17 Statistical analyses

Statistical analyses were conducted using SPSS version 22 statistical software. In vitro experiments were independently repeated three times. In the case of in vivo experiments, six samples were utilised in each group for statistical analysis. A two‐tailed Student's *t*‐test was employed for comparisons between two groups, while one‐way ANOVA, followed by Bonferroni's post hoc comparisons, was used for comparisons involving three or more groups. *p*‐values below 0.05 were considered statistically significant.

## 3 RESULTS

### 3.1 FBLN1 enhances the osteogenic differentiation and bone regeneration ability of WJCMSCs

**FIGURE 1:**
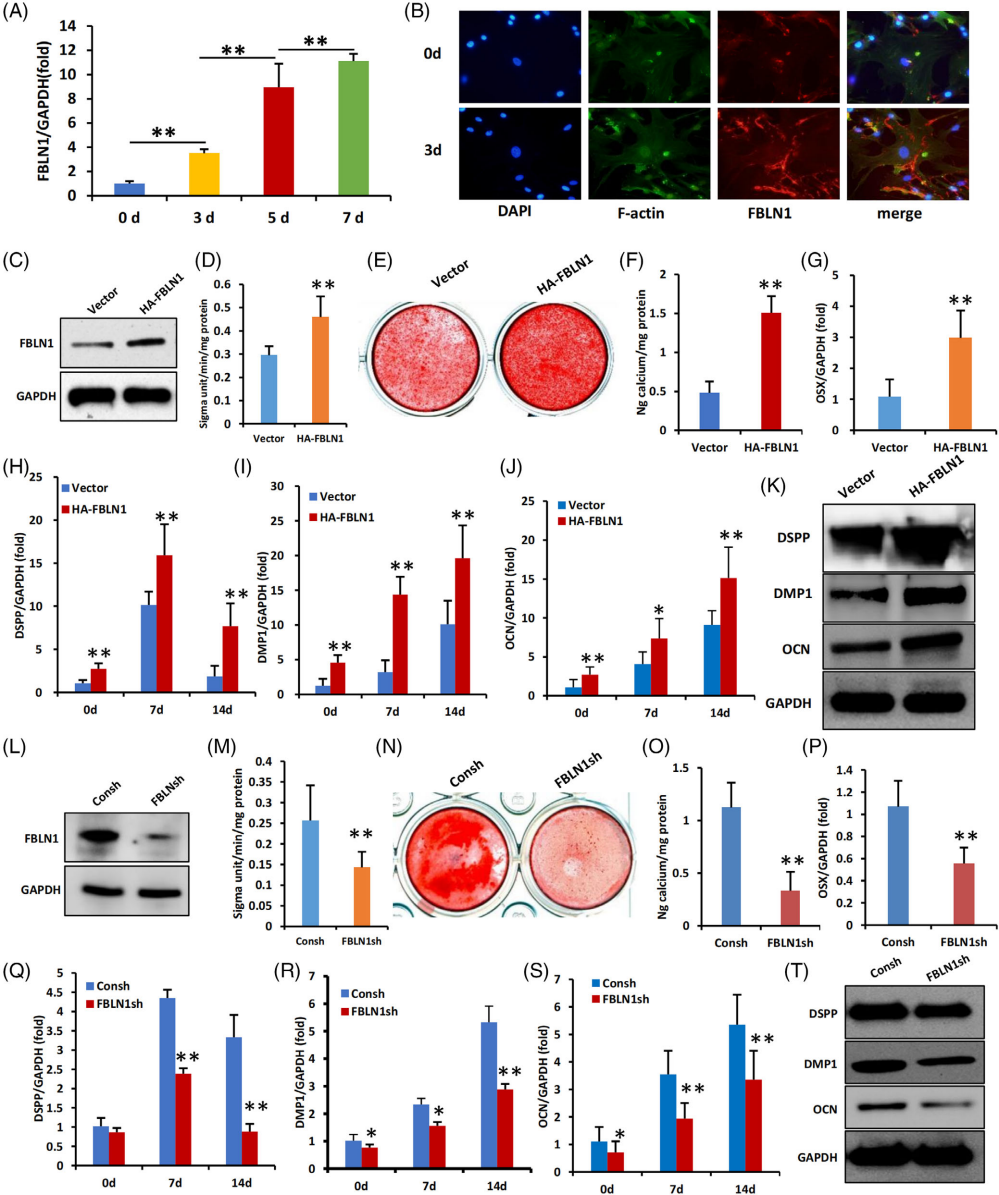
FBLN1 promotes osteogenic differentiation of WJCMSCs in vitro. (A) qPCR showed the expression of FBLN1 on 0, 3, 5, 7 days in WJCMSCs after mineralization induction. (B) Immunofluorescence staining of FBLN1 in WJCMSCs 3 days after mineralization induction. (C) Western Blot confirmed the overexpression of FBLN1 in WJCMSCs. (D) FBLN1 overexpression increased ALP activity in WJCMSCs. (E, F) Alizarin Red staining and quantification of Alizarin Red results. (G–K) qPCR showed that overexpression of FBLN1 upregulated the RNA expression levels of OSX (G), DSPP (H), DMP1 (I) and OCN (J) in WJCMSCs. (K) Overexpression of FBLN1 increased the protein levels of DSPP, DMP1 and OCN in WJCMSCs on 14 days after mineralization induction. (L) Western Blot verified that FBLN1 was knocked down in WJCMSCs. (M) knockdown of FBLN1 decreased ALP activity in WJCMSCs. (N, O) Alizarin Red staining and quantification of Alizarin Red results. (P–T) qPCR showed that knockdown of FBLN1 downregulated the RNA expression levels of OSX (P), DSPP (Q), DMP1 (R) and OCN (S) in WJCMSCs. (T) knockdown of FBLN1 decreased the protein levels of DSPP, DMP1 and OCN in WJCMSCs on 14 days after mineralization induction. GAPDH was used as the internal control. Data were presented as mean + SD (*n* = 3). Statistical analysis was performed using Student's *t*‐test. *p ≤ 0.05. **p ≤ 0.01.

WJCMSCs were cultured in mineralization medium for 7 days, and the results demonstrated a time‐dependent increase in the expression of FBLN1 during the process of osteogenic differentiation of WJCMSCs (Figure 1A). Immunofluorescence staining revealed that the protein level of FBLN1 was upregulated in WJCMSCs after three days of mineralization induction (Figure 1B). To investigate the functions of FBLN1 on osteogenic differentiation of WJCMSCs, HA‐FBLN1 expression construct was transfected into WJCMSCs by virus infection and confirmed by Western blot (Figure 1C). Results demonstrated that FBLN1 overexpression promoted the osteogenic differentiation of WJCMSCs as revealed by higher ALP activity and more alizarin red staining in FBLN1 overexpression group on day 3 and day 14, respectively, after mineralization induction in WJCMSCs (Figure 1D–F). In addition, overexpression of FBLN1 notably increased the expression of OSX, DSPP, DMP1, and OCN in WJCMSCs as detected by qPCR and Western blot (Figure 1G–K).

We also knockdown the expression of FBLN1 with a short hairpin RNA (shRNA) to further confirm the its functions in WJCMSCs (Figure 1L). Results showed that knockdown of FBLN1 decreased the ALP activity and alizarin red staining on day 3 and day 14, respectively, after mineralization induction in WJCMSCs, indicating the impaired osteogenic differentiation ability of FBLN1‐knockdown WJCMSCs cells (Figure 1M–O). Furthermore, the expression levels of OSX, DSPP, DMP1, and OCN were reduced in FBLN1‐knockdown WJCMSCs as revealed by qPCR and Western blot (Figure 1P–T).

**FIGURE 2:**
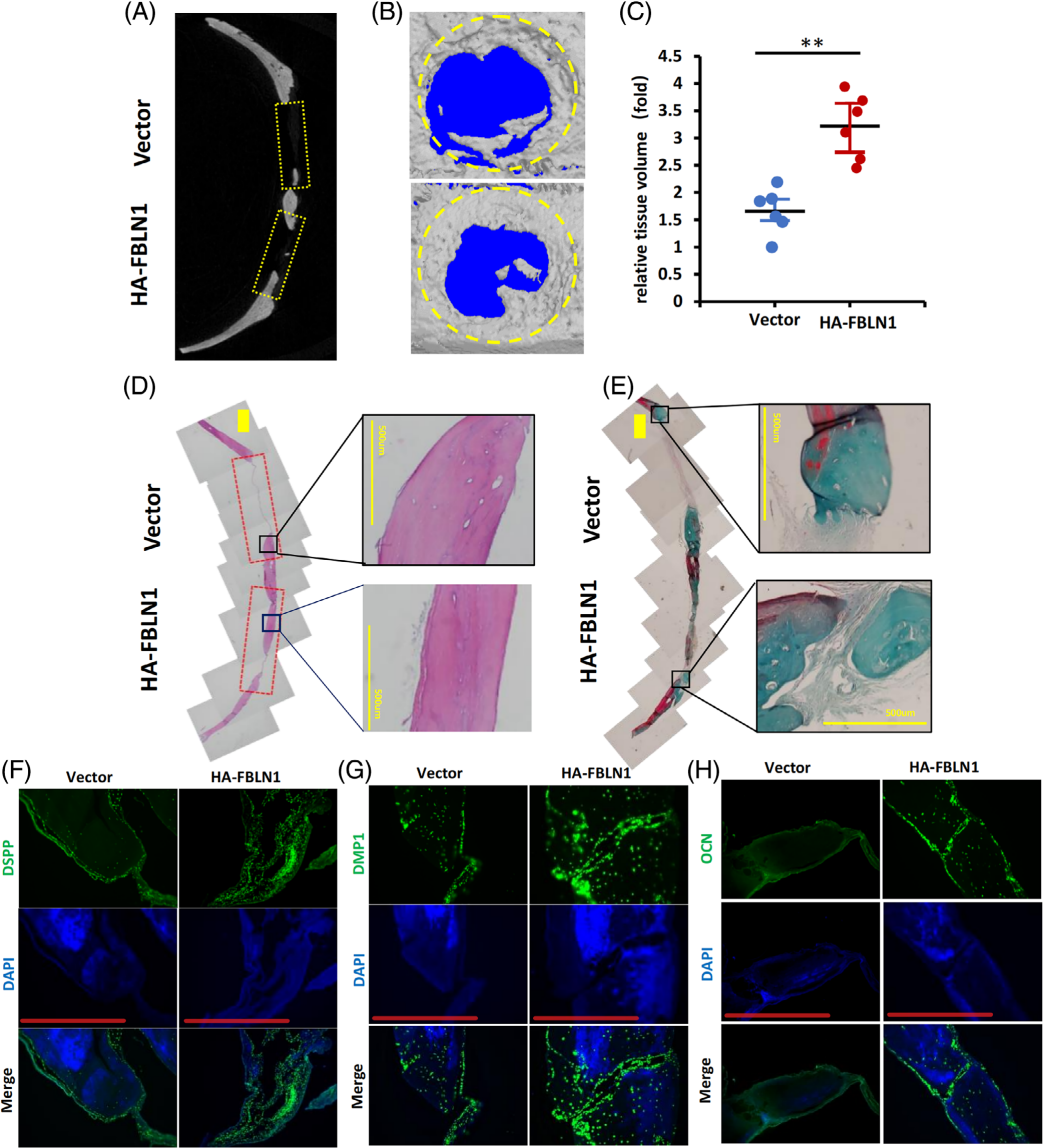
FBLN1 enhances the bone regeneration of WJCMSCs in vivo. (A, B) Representative micro‐CT and the 3D reconstructed micro‐CT images of the critical‐sized defect in the WJCMSCs/Vector group and the WJCMSCs/HA‐FBLN1 group. (C) The relative tissue volume levels were quantified by 3D volumetric analysis. (D) Histological sections images of the critical‐sized defect, as shown by an H&E staining assay, the WJCMSCs/HA‐FBLN1 group had significantly more new bone formation than the WJCMSCs/Vector group (Bar: 1 mm or 500 μm). (E) Masson staining assay showed that the amount of collagenous fibre and the area of new bone formation in the WJCMSCs/HA‐FBLN1 group was greater than that in the WJCMSCs/Vector group (Bar: 1 mm or 500 μm). (F–H) Immunofluorescence staining showed the DSPP, DMP1 and OCN expression (Bar: 500 μm). The student's *t*‐test was performed to determine statistical significance. All error bars represent the SD. (*n* = 6). **p ≤ 0.01.

We next examine whether FBLN1 affects in situ bone regeneration in vivo, Calvaria critical‐sized defects model (CSD) in nude rats were used. WJCMSCs/Vector or WJCMSCs/HA‐FBLN1 cells were transplanted into the defect site. The regeneration area of bone tissue in WJCMSCs/HA‐FBLN1 group was significantly more than in the WJCMSCs/Vector group in the defect area as revealed by micro‐CT (Figure 2A). Through 3D volume analysis, we found that the volume level of regenerated bone tissue in WJCMSCs/HA‐FBLN1 group was much higher than that in WJCMSCs/Vector group (Figure 2B,C). New bone formation was significantly higher in the WJCMSCs/HA‐FBLN1 group compared to the WJCMSCs/Vector group revealing by H&E staining results (Figure 2D). In the WJCMSCs/HA‐FBLN1 group, collagen fibres and new bone formation were significantly greater than in the WJCMSCs/Vector group based on Masson staining results (Figure 2E). The DSPP, DMP1, and OCN expression were significantly higher in the WJCMSCs/HA‐FBLN1 group than in the WJCMSCs/Vector group, similar to the results from the in vitro study (Figure 2F–H).

### 3.2 Mutation of m^6^A site in FBLN1 3′UTR enhanced the stability of FBLN1 mRNA

**FIGURE 3:**
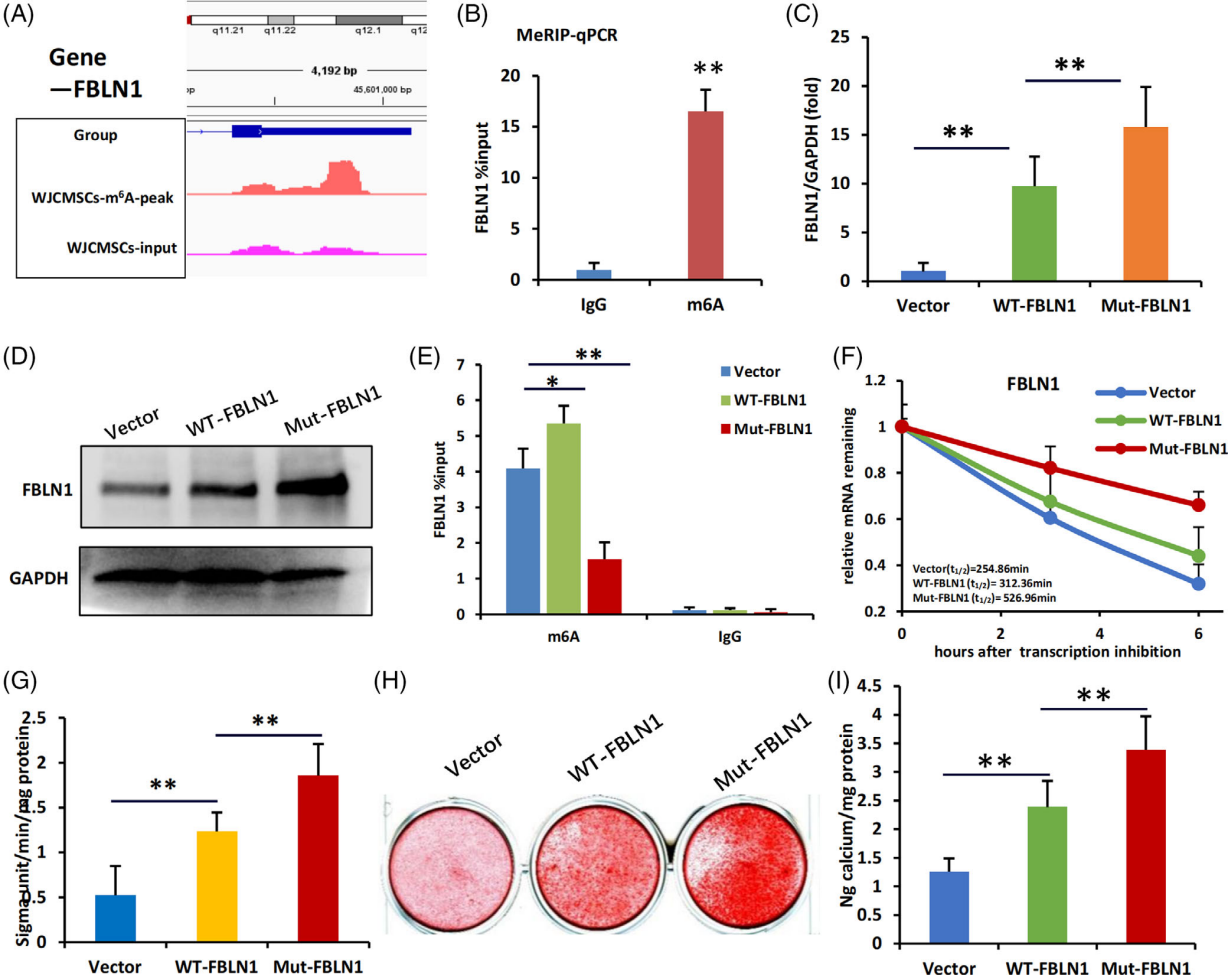
Mutating the m^6^A modification site within FBLN1 3′UTR enhance the stability of FBLN1 mRNA. (A) m^6^A peaks were enriched in 3′UTRs of FBLN1 genes from MeRIP‐seq data in WJCMSCs. (B) MeRIP‐qPCR analysis showed Enrichment of m^6^A‐modified FBLN1 in WJCMSCs. (C) qPCR was used to measure the expression of FBLN1 in Vector, WT‐FBLN1 and Mut‐FBLN1groups. (D) The Western Blot results showed the expression of FBLN1 in Vector, WT‐FBLN1 and Mut‐FBLN1 groups. (E) MeRIP‐qPCR analysis showed enrichment of m^6^A‐modified FBLN1 in Vector, WT‐FBLN1 and Mut‐FBLN1 groups. (F) Actinomycin D chase assay showed that the mRNA stability of FBLN1 in Vector, WT‐FBLN1 and Mut‐FBLN1 groups after Actinomycin D treatment (5 μg/mL) for 3 and 6 h. (G) ALP activity analysis results showed in Vector, WT‐FBLN1 and Mut‐FBLN1 groups. (H, I) Alizarin red staining and quantification analysis results showed in Vector, WT‐FBLN1 and Mut‐FBLN1 groups. GAPDH was used as the internal control. Student's *t*‐test and One‐way ANOVA were performed to determine statistical significance. All error bars represent the SD (*n* = 3). *p ≤ 0.05. **p ≤ 0.01.

We next studied the molecular mechanism regulating FBLN1 expression in WJCMSCs. Using m^6^A bioinformatics algorithms, we identified potential m^6^A sites in FBLN1 mRNA (Figure S1). MeRIP‐seq analysis (Table S3) and m^6^A‐RIP‐qPCR confirmed m^6^A methylation site in the 3′UTR regions of FBLN1 mRNA in WJCMSCs (Figure 3A,B). We transfected WT‐FBLN1, Mut‐FBLN1 (m^6^A site defective) or empty vector into WJCMSCs and found that disruption of the m^6^A site of FBLN1 showed stronger overexpression efficiency of FBLN1 compared to that of the WT‐FBLN1 group (Figure 3C,D). MeRIP‐qPCR showed that m^6^A modification was the highest in the WT‐FBLN1 group, followed by the Vector group, and was the lowest in the Mut‐FBLN1 group (Figure 3E). Actinomycin D assay showed greater stability of FBLN1 mRNA in Mut‐FBLN1 group compared to WT‐FBLN1 group (Figure 3F). Functional analysis revealed the stronger osteogenic differentiation ability in the WJCMSCs/Mut‐FBLN1 group analysing by ALP activity and ARS staining compared to the WJCMSCs/WT‐FBLN1 group (Figure 3G–I). These findings indicate that a mutation at the m^6^A site within the FBLN1 3′UTR can enhance the stability of FBLN1 mRNA and ultimately lead to the enhanced FBLN1 function for osteogenic differentiation of WJCMSCs.

### 3.3 miR‐615‐3p negatively regulated FBLN1 in a 3′UTR‐dependent manner

Several studies propose that m^6^A modification of target mRNA may play a pivotal role in the regulation of miRNA functionality. Hence, we aimed to investigate whether there are specific miRNAs that regulate the 3′UTR of FBLN1 through m^6^A modification regulation. In our study, we initially analysed the predictive results of microRNA (Table S2) and subsequently identified microRNA‐binding sites in close proximity to the m^6^A modification site. Furthermore, previous literature has reported on the significant role played by miR‐615‐3p.^29^ Consequently, we hypothesised that miR‐615‐3p potentially regulates FBLN1.

**FIGURE 4:**
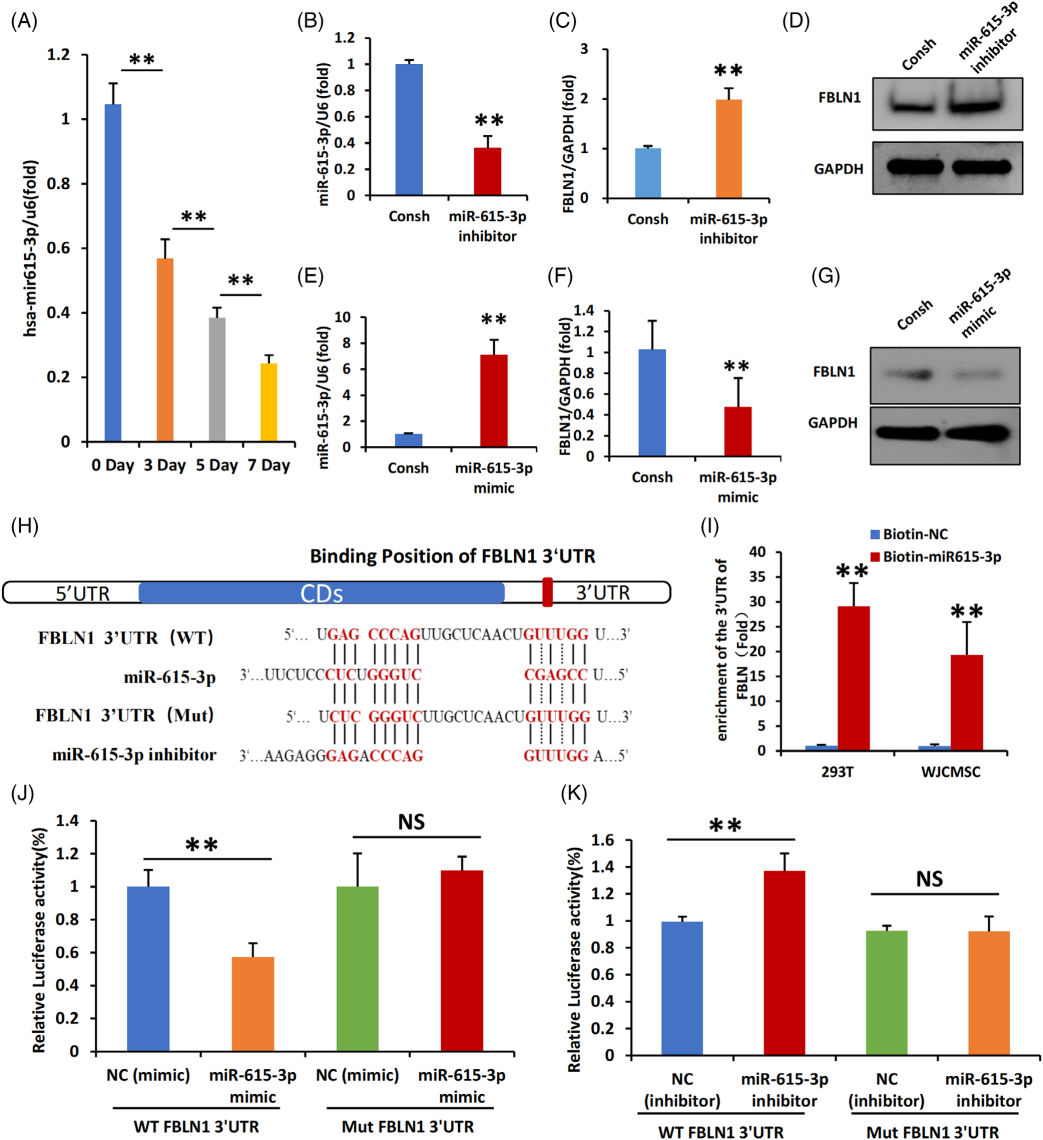
miR‐615‐3p regulates FBLN1 by directly targeting the 3′UTR of FBLN1. (A) The qPCR results showed the expression of miR‐615‐3p on 0, 3, 5 and 7 days in WJCMSCs after mineralization induction. (B) The qPCR results showed that miR‐615‐3p was knocked down in WJCMSCs. (C, D) qPCR (C) and Western blot (D) results showed that FBLN1 expression was increased in miR‐615‐3p depleted WJCMSCs. (E) The qPCR results showed that miR‐615‐3p was overexpression in WJCMSCs. (F, G) qPCR (F) and Western blot results (G) showed that FBLN1 expression was inhibited in miR‐615‐3p overexpressed WJCMSCs. (H) The predicted miR‐615‐3p binding site in the 3′UTR of FBLN1 and the corresponding mutant binding site are shown. (I) Enrichment of immunoprecipitated biotin‐labelled miR‐615‐3p RNA in 293 T cells and WJCMSCs. (J, K) Relative luciferase activities of reporters containing the 3′UTR of FBLN1 (WT‐FBLN1) or mutant construct in 293 T cells 48 hr after cotransfection with miR‐615‐3p mimic (J) or miR‐615‐3p inhibitor (K). Firefly luciferase activity was normalized to control Renilla luciferase activity. Student's *t*‐test and One‐way ANOVA were performed to determine statistical significance. GAPDH and U6 were used as the internal control. All error bars represent the SD (*n* = 3). **p ≤ 0.01.

Therefore, our continuous monitoring of miR‐615‐3p expression revealed a time‐dependent decrease in its expression levels during the mineralization induction process in WJCMSCs (Figure 4A). Then, knockdown of miR‐615‐3p increased the expression of FBLN1 at both RNA and protein levels, while overexpression of miR‐615‐3p suppressed the expression of FBLN1 in WJCMSCs (Figure 4B–G). The potential binding sites of miR‐615‐3p in the 3′UTR of FBLN1 were predicted using miRNA bioinformatics algorithms software (Figure 4H). To verify the regulation of miR‐615‐3p on FBLN1 expression, we initially analyse the interaction between miR‐615‐3p and FBLN1 RNA. Biotin‐labelled miR‐615‐3p was used to co‐precipitation FBLN1 3′UTR RNA in 293 T and WJCMSCs and the results suggested that miR‐615‐3p interacted with FBLN1 3′UTR (Figure 4I). Luciferase Reporter Gene Assay was further used to confirm the interaction. We cloned the wild‐type or mutated miR‐615‐3p‐binding sites in the FBLN1 3′UTR region into a luciferase reporter gene vector for Luciferase Reporter Gene Assay. Results showed that miR‐615‐3p mimic suppressed the luciferase activity of WT‐FBLN1 3′UTR group, but not in the Mut‐FBLN1 3′UTR group (Figure 4J). Consistently, miR‐615‐3p inhibitor upregulated the luciferase activity of WT‐FBLN1 3′UTR group, not in the Mut‐FBLN1 3′UTR group (Figure 4K). These results indicated that miR‐615‐3p could directly target to the binding site of FBLN1 and downregulate its expression.

### 3.4 miR‐615‐3p inhibits the osteogenic differentiation and bone regeneration ability of WJCMSCs

**FIGURE 5:**
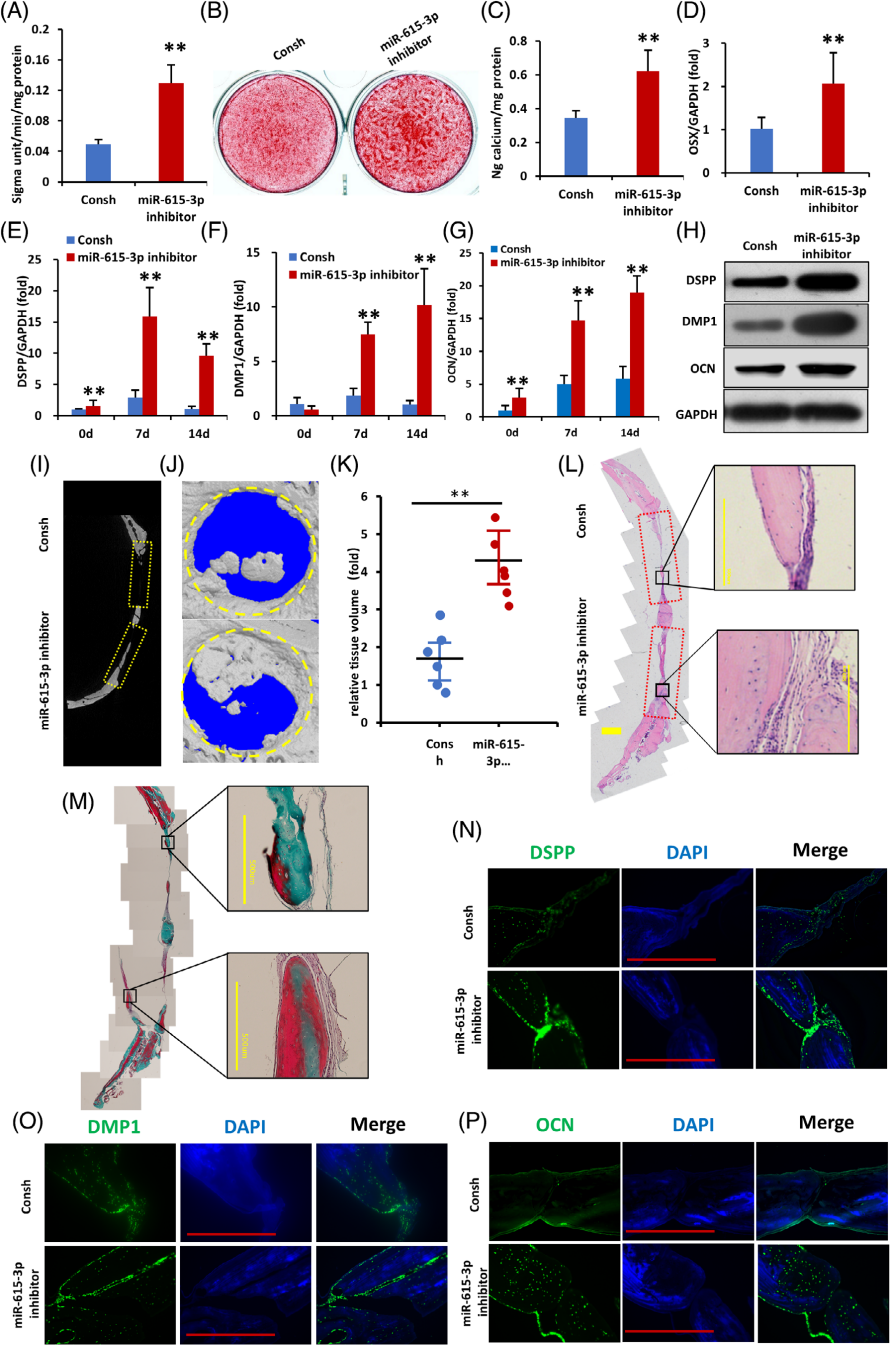
miR‐615‐3p directly targets FBLN1's 3′UTR, negatively regulating osteogenic differentiation of bone regeneration ability of WJCMSCs. (A) miR‐615‐3p knockdown increased ALP activity in WJCMSCs. (B, C) Alizarin red staining and quantification analysis. (D) qPCR results showed that knockdown of miR‐615‐3p upregulated the expression of OSX in WJCMSCs. (E–G) qPCR results showed that knockdown of miR‐615‐3p increased DSPP (E), DMP1 (F) and OCN (G) in WJCMSCs after mineralization induction. (H) The protein expression levels of DSPP, DMP1 and OCN on day 14 after mineralization induction. (I, J) Representative micro‐CT and the 3D reconstructed micro‐CT images of the critical‐sized defect in the WJCMSCs/Consh group and the WJCMSCs/miR‐615‐3p‐inhibitor group. (K) The relative tissue volume levels were quantified by 3D volumetric analysis. (L) Histological sections images of the critical‐sized defect, as shown by an H&E staining assay, the WJCMSCs/ miR‐615‐3p‐inhibitor group had significantly more new bone formation than the WJCMSCs/Consh group (Bar: 1 mm or 500 μm). (M) Masson staining assay showed that the amount of collagenous fibre and the area of new bone formation in the WJCMSCs/miR‐615‐3p‐inhibitor group was greater than that in the WJCMSCs/Consh group (Bar: 1 mm or 500 μm). (N–P) Immunofluorescence staining showed the DSPP, DMP1 and OCN expression (Bar: 500 μm). GAPDH served as the internal control. Student's *t*‐test was performed to determine statistical significance. All error bars represent the SD (*n* = 3 or 6). **p ≤ 0.01.

To investigate the function of miR‐615‐3p during osteogenic differentiation in WJCMSCs, loss‐of‐function studies were conducted in WJCMSCs using lentiviral miR‐615‐3p inhibitors. Depletion of miR‐615‐3p enhanced the osteogenic differentiation ability of WJCMSCs as revealed by higher ALP activity and more alizarin red staining in miR‐615‐3p inhibitor group on day 3 and day 14, respectively, after mineralization induction in WJCMSCs (Figure 5A–C) and the upregulated expression of OSX, DSPP, DMP1 and OCN as revealed by qPCR and Western blot (Figure 5D–H). On the contrary, gain‐of‐function studies using miR‐615‐3p mimics exhibited opposite results. Overexpression of miR‐615‐3p in WJCMSCs inhibited the osteogenic differentiation ability of WJCMSCs as revealed by decreased ALP activity and alizarin red staining in miR‐615‐3p mimics group on day 3 and day 14, respectively, after mineralization induction in WJCMSCs (Figure S2A–C) and downregulated expressions of OSX, DMP‐1, DSPP, and OCN (Figure S2D–H).

To further verify the bone regeneration function of miR‐615‐3p, the CSD model in rats was used. WJCMSCs/miR‐615‐3p inhibitor group regenerated more bone tissue than WJCMSCs/Consh group according to micro‐CT results (Figure 5I). The three‐dimensional image reconstructed by micro‐CT was next performed. The healing percentage was greater in the WJCMSCs/miR‐615‐3p‐inhibitor group than that of the control group (Figure 5J,K). Histological examination demonstrated that in the WJCMSCs/miR‐615‐3p inhibitor group, there was an increased formation of new bone tissue, which appeared to be both more abundant and thicker, especially in the central and peripheral regions of the defect area, as compared to the WJCMSCs/Consh group (Figure 5L). Based on Masson staining, collagen fibres and new bone formation were significantly greater in the WJCMSCs/miR‐615‐3p‐inhibitor group than in the WJCMSCs/Consh group (Figure 5M). The DSPP, DMP1, and OCN expression were significantly higher in the WJCMSCs/ miR‐615‐3p‐inhibitor group than in the WJCMSCs/Consh group, similar to the results from the in vitro study (Figure 5N–P). To sum up, these findings indicate that miR‐615‐3p inhibited the osteogenic differentiation and in situ bone regeneration of WJCMSCs.

### 3.5 The mutation of miR‐615‐3p‐binding sites or m^6^A sites in the 3′UTR of FBLN1 affect the FBLN1 and WJCMSC function

**FIGURE 6:**
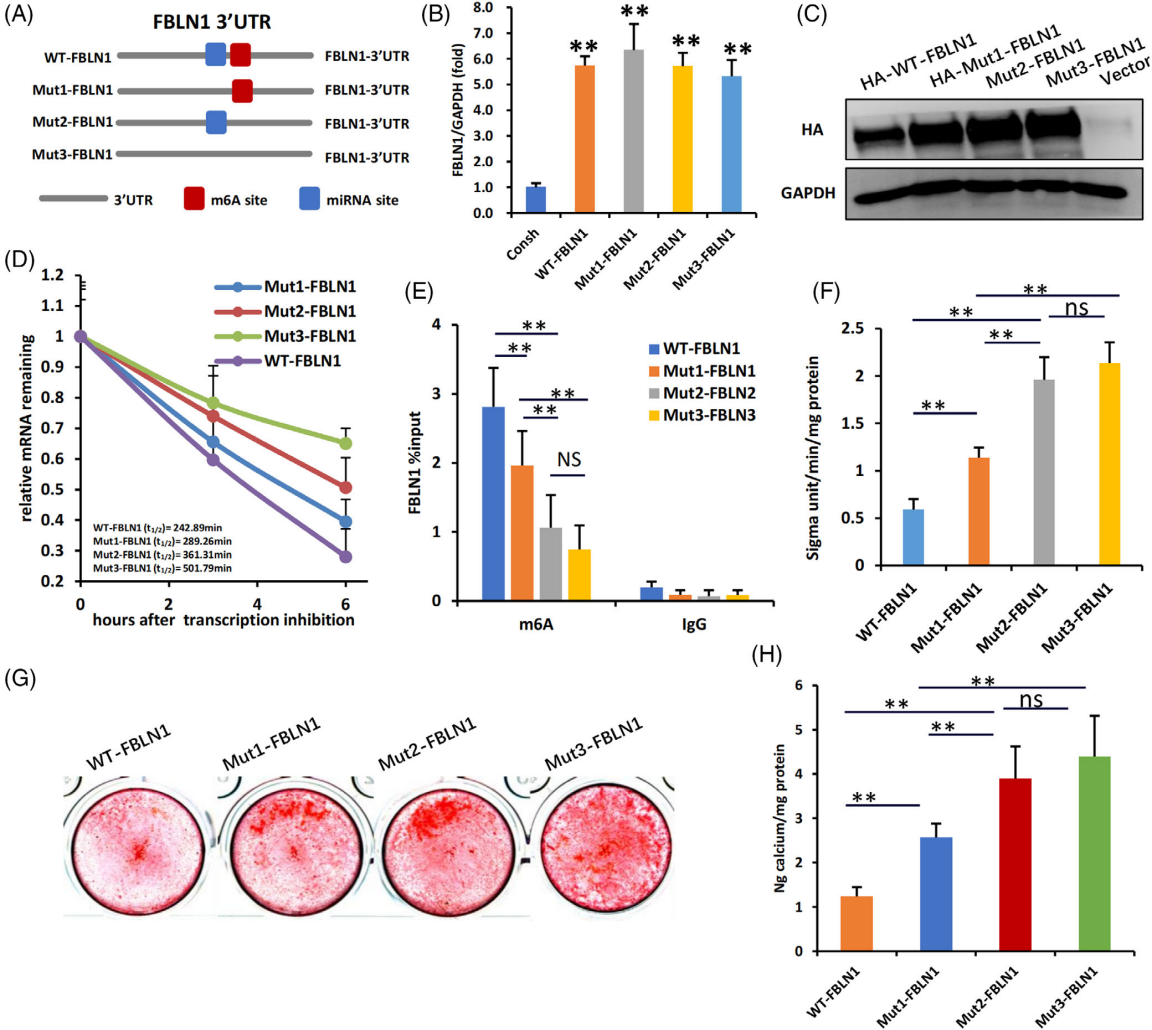
The mutation of miR‐615‐3p binding sites or m^6^A motif sites in the 3′UTR of FBLN1 affect the FBLN1 and WJCMSC function. (A) Schematic representation of miR‐615‐3p binding sites and m^6^A motif sites in the 3′UTR of FBLN1, and wild‐type (WT) and mutant type (MUT1, MUT2, MUT3) lentiviruses constructed based on these sites. (B) qPCR was used to measure the expression of FBLN1 in cellular DNA. (C) The Western Blot results showed the expression of HA‐tag in WT and MUT groups. (D) Actinomycin D chase assay showed that the mRNA stability of FBLN1 in WT and MUT groups after Actinomycin D treatment (5 μg/mL) for 3 and 6 h. (E) MeRIP‐qPCR analysis showed enrichment of m^6^A‐modified FBLN1 in WT and MUT groups. (F) ALP activity analysis results showed in WT and MUT groups. (G, H) Alizarin red staining and quantification analysis results showed in WT and MUT groups. One‐way ANOVA was performed to determine statistical significance. All error bars represent the SD.(*n* = 3).**p ≤ 0.05.

To investigate the potential relationship between m^6^A modification, miR615‐3p, and FBLN1 expression, we constructed FBLN1 overexpression vectors with mutated miR615‐3p‐binding site (Mut1‐FBLN1), m^6^A modification site (Mut2‐FBLN1), or both sites simultaneously (Mut3‐FBLN1) (Figure 6A). Results found that transfected cells with these four kinds of FBLN1 overexpression vectors showed similar DNA expression levels of FBLN1 (Figure 6B), however, the protein levels of FBLN1 in mutant groups were higher than that of the WT‐FBLN1 group, indicating that disruption of the m^6^A site or the miR615‐3p‐binding site promoted FBLN1 protein expression in a posttranscriptional regulation manner (Figure 6C). The Actinomycin D experiments revealed a reduction in the degradation of FBLN1 mRNA upon disruption of either the m^6^A site or miR615‐3p‐binding site. However, simultaneous disruption of both sites resulted in the slowest degradation of FBLN1 mRNA (Figure 6D). MeRIP‐qPCR revealed that mutation of either the m^6^A site or miR615‐3p‐binding site reduced m^6^A level of FBLN1 mRNA, and the reduction in miR615‐3p‐binding site mutation group is higher than that in m^6^A site mutation group (Figure 6E). In addition, all three FBLN1 mutation groups showed higher ALP activity (Figure 6F) and stronger alizarin red staining (Figure 6G,H) compared to the WT‐FBLN1 group. Among the three mutation groups, the osteogenic differentiation of Mut2‐FBLN1 and Mut3‐FBLN1 groups was stronger than that of Mut1‐FBLN1 group assayed by ALP activity and alizarin red staining (Figure 6F–H). It noted that there was no statistical difference between the Mut2‐FBLN1 and Mut3‐FBLN1groups. This suggests that disruption of the m^6^A site or the miR615‐3p‐binding site promoted mineralization ability of FBLN1, and miR615‐3p regulate the functions of FBLN1may through an m^6^A‐dependent model.

### 3.6 YTHDF2 and miR‐615‐3p protein‐RNA complex negatively regulates the stability of FBLN1

**FIGURE 7:**
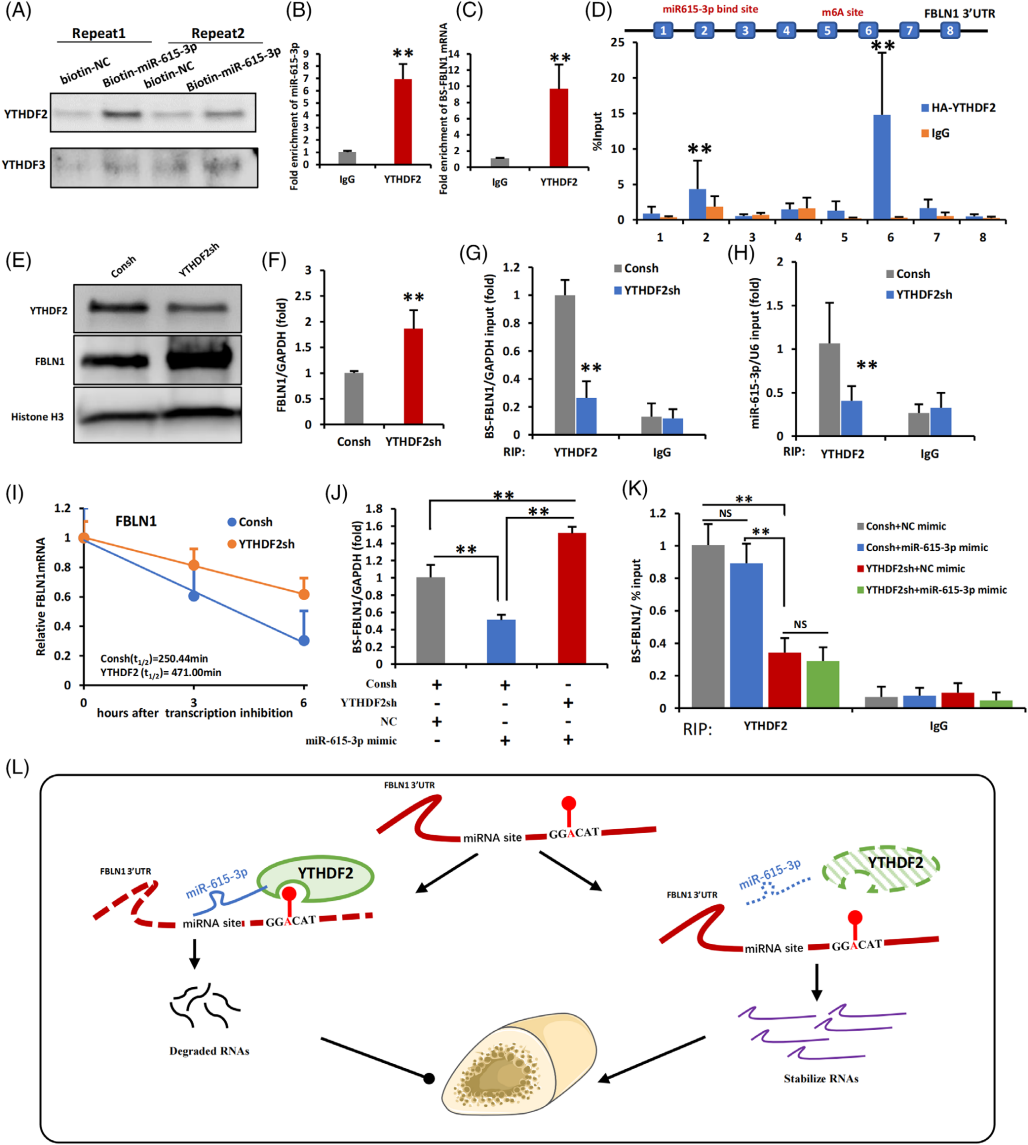
miR‐615‐3p and YTHDF2 combined to form a complex and associated with FBLN1 3′UTR. (A) Streptavidin‐biotin RNA‐protein binding assay results showed that YTHDF2 could co‐immunoprecipitates with biotin‐labelled miR‐615‐3p. (B) RIP results showed that YTHDF2 protein could co‐immunoprecipitates with miR‐615‐3p. (C) RIP results showed that YTHDF2 protein could co‐immunoprecipitates with 3′UTR of FBLN1 mRNA. (D) CLIP‐RT‐qPCR monitoring binding of HA‐YTHDF2 to 3′UTR of FBLN1 in WJCMSCs. Schematic illustration of PCR primers designed at the 3′UTR of FBLN1. Precipitated RNA was analysed by qPCR using the indicated primers. (E) The western blot results showed that YTHDF2 was knocked down and FBLN1 was upregulated in WJCMSCs/YTHDF2sh group. Histone H3 was used as a loading control. (F) qPCR results showed that FBLN1 was upregulated in WJCMSCs/YTHDF2sh group. (G) RIP results showed that FBLN1 mRNA could co‐immunoprecipitate with less YTHDF2 protein in WJCMSC/YTHDF2sh group. (H) RIP results showed that miR‐615‐3p could co‐immunoprecipitate with less YTHDF2 protein in WJCMSC/YTHDF2sh group. (I) Actinomycin D chase assay showed that the mRNA stability of FBLN1 in WJCMSCs/YTHDF2sh group was better than that in WJCMSCs/Consh group after Actinomycin D treatment (5 μg/mL) for 3 and 6 h. (J) In WJCMSCs/YTHDF2sh group and WJCMSCs/Consh group, miR‐615‐3p mimics and control mimics were added to detect the expression of FBLN1 by qPCR. (K) RIP results showed that FBLN1 mRNA could co‐immunoprecipitate with less YTHDF2 protein in WJCMSC/YTHDF2sh group, the addition of miR‐615‐3p mimics did not change the binding state of YTHDF2 protein and FBLN1 mRNA. (L) Working model of the regulation for the protein‐RNA complex of miR‐615‐3p and YTHDF2 on the FBLN1 stability. Student's *t*‐test and One‐way ANOVA were performed to determine statistical significance. All error bars represent the SD (*n* = 3). **p ≤ 0.01.

To elucidate the proteins involved in FBLN1 mRNA decay, we employed a biotin‐labelled miR‐615‐3p RNA immunoprecipitation (RIP) assay and iTRAQ analysis, and found that the YTH family members YTHDF2 and YTHDF3 were coprecipitated with miR615‐3p (Table S4). Biotin‐RNA pull‐down assays validated the specific interaction between miR‐615‐3p and YTHDF2, rather than YTHDF3, as confirmed by our study (Figure 7A). By RIP assays, we revealed that YTHDF2 exhibited binding affinity towards both miR615‐3p and FBLN1 3′UTR (Figure 7B,C). Furthermore, CLIP‐qPCR experiments showed that YTHDF2 could bind to both the miR‐615‐3p‐binding site and the m^6^A‐modified site simultaneously (Figure 7D). WJCMSCs with YTHDF2 knockdown expressed more FBLN1 mRNA and protein (Figure 7E,F). Based on these results, it is likely that YTHDF2 is involved in posttranscriptional regulation of FBLN1. We designed a primer named ‘BS‐FBLN1’, based on the binding site of miR‐615‐3p and the m^6^A‐modified region within FBLN1, and used this primer to detect the binding of the two sites. Next, RIP experiments were conducted to examine the binding relationship between miR‐615‐3p, FBLN1 3′UTR and YTHDF2. The findings indicated that in the YTHDF2sh/WJCMSCs group, the interaction between YTHDF2 and FBLN1 3′UTR was notably reduced, accounting for approximately 74% reduction in binding compared to the control group (Figure 7G), and the binding of YTHDF2 to miR‐615‐3p was also reduced by approximately 62% in the YTHDF2sh/WJCMSCs group (Figure 7H). Actinomycin D assays indicated that YTHDF2 knockdown extended the stability of FBLN1 mRNA, suggesting that Knockdown of YTHDF2 suppressed FBLN1 mRNA degradation, increased FBLN1 expression (Figure 7I). Moreover, to examine the priority of miR‐615‐3p versus YTHDF2 in regulating the FBLN1 3′UTR. The miR‐615‐3p mimic was added to WJCMSCs/YTHDF2sh, and the expression level of FBLN1 was detected by qPCR. The results showed that the FBLN1 expression was restored after adding miR‐615‐3p mimic (Figure 7J). This suggests that the absence of YTHDF2 eliminates the inhibitory effect of miR‐615‐3p on FBLN1. Therefore, we conducted a RIP experiment to confirm the combination state of the three in this state. The results showed that miR‐615‐3p could not bind to more FBLN1 3′UTR when YTHDF2 was absent (Figure 7K).

On the other hand, the overexpression of YTHDF2 resulted in an increased binding affinity of YTHDF2 to both the FBLN1 3′UTR and miR‐615‐3p, ultimately leading to a reduction in mRNA and protein levels of FBLN1 by accelerating its degradation within WJCMSCs (Figure S3A–E). The upregulation of YTHDF2 counteracted the stimulatory impact exerted by the miR‐615‐3p inhibitor on FBLN1 expression. (Figure S3F). RIP experiment also showed that that inhibiting miR‐615‐3p promoted the binding of YTHDF2 protein to FBLN1 mRNA (Figure S3G). Additionally, YTHDF2 expression was unaffected by miR‐615‐3p mimic (Figure S4A), and the ALP activity and ARS staining showed no significant differences in WJCMSCs with YTHDF2 knockdown after adding miR‐615‐3p mimics (Figure S4B,C). Similar conclusions were reached through experiments with YTHDF2 overexpression in WJCMSCs (Figure S4D–F). The miRNA‐loaded AGO forms the targeting module of the miRNA‐induced silencing complex (miRISC). Therefore, immunofluorescence staining was performed first, and the results showed that YTHDF2 and AGO2 were co‐localised to WJCMSCs (Figure S5A). Further, Co‐IP experiments revealed that YTHDF2 could interact with AGO2 in an RNA‐dependent manner (Figure S5B), and miR‐615‐3p had no effect on this interaction (Figure S5C).

Our study demonstrates that YTHDF2 can bind with the specific m^6^A site on the 3′UTR of FBLN1 mRNA, and miRNA‐615‐3p recognises and binds to the 3′UTR region of FBLN1 mRNA. YTHDF2 can form a complex with miRNA‐615‐3p, destroying the FBLN1 mRNA stability and ultimately leading to decreased FBLN1 expression (Figure 7L).

### 3.7 YTHDF2 inhibits the osteogenic differentiation of WJCMSCs

**FIGURE 8:**
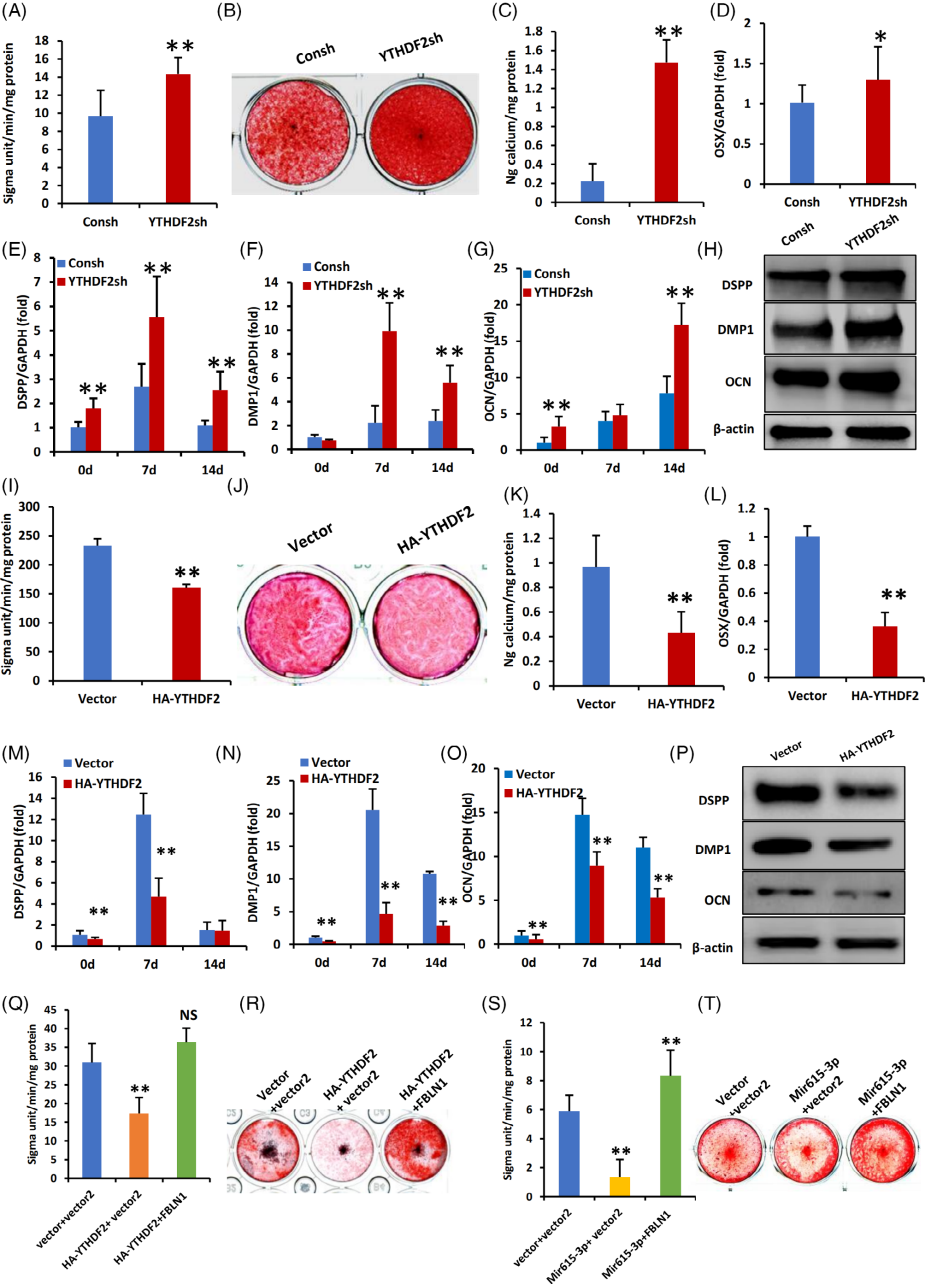
YTHDF2 Inhibits osteogenic differentiation in WJCMSCs. (A) YTHDF2 knockdown increased ALP activity in WJCMSCs. (B, C) Alizarin red staining and analysis results. (D) qPCR results showed that knockdown of YTHDF2 upregulated OSX in WJCMSCs. (E–G) qPCR results showed that knockdown of YTHDF2 upregulated DSPP(E), DMP1 (F) and OCN(G). (H) knockdown of YTHDF2 decreased the protein levels of DSPP, DMP1 and OCN at 14 days after mineralization induction. (I) YTHDF2 overexpression decreased ALP activity in WJCMSCs. (J, K) Alizarin red staining results. (L) qPCR results showed that overexpression of YTHDF2 downregulated OSX in WJCMSCs. (M–O) qPCR results showed that overexpression of YTHDF2 downregulated DSPP (M), DMP1 (N) and OCN(O). (P) Overexpression of YTHDF2 decreased the protein levels of DSPP, DMP1 and OCN at 14 days after mineralization induction. (Q, R) ALP activity and Alizarin red staining results showed that FBLN1 could rescue the reduced mineralization function caused by YTHDF2 in WJCMSC. (S, T) ALP activity and Alizarin red staining results showed that FBLN1 could rescue the reduced mineralization function caused by miR‐615‐3p in WJCMSC. β‐actin was used as a loading control. GAPDH was used as an internal control. Student's *t*‐test and One‐way ANOVA were performed to determine statistical significance. All error bars represent the SD (*n* = 3). *p ≤ 0.05. **p ≤ 0.01.

Then, we aim to elucidate the role of YTHDF2 in promoting osteogenic differentiation in WJCMSCs. The findings demonstrated that depletion of YTHDF2 significantly augmented the osteogenic differentiation potential of WJCMSCs, as evidenced by elevated ALP activity and enhanced mineralization capacity (Figure 8A–C). The OSX, DMP1, DSPP, and OCN expression was upregulated (Figure 8D–H). In contrast, the overexpression of YTHDF2 in WJCMSCs was found to impede the osteogenic differentiation potential, as evidenced by a reduction in ALP activity and calcium mineralization (Figure 8I–K), and downregulated expression of OSX, DMP‐1, DSPP, and OCN (Figure 8L–P).

Since both YTHDF2 and miR‐615‐3p exerted inhibitory effects on the osteogenic differentiation potential of WJCMSCs, we added FBLN1 to the WJCMSCs/YTHDF2 group and WJCMSCs/miR‐615‐3p group, respectively, to perform rescue experiments. The results showed that overexpression of FLBN1 to the WJCMSCs/YTHDF2 group (Figure 8Q,R) and WJCMSCs/miR‐615‐3p group (Figure 8S,T), respectively, could save the osteogenic differentiation ability of the WJCMSCs detected ALP activity and mineralization ability.

## 4 DISCUSSION

FBLN1 is known to deposit in the ECM and associate closely with osteoblast maturation. However, the precise effects and underlying regulatory mechanisms of FBLN1 on bone regeneration are still to be elucidated. Our findings demonstrate that in vitro, FBLN1 possesses the ability to augment the osteogenic differentiation of WJCMSCs, substantiated by elevated ALP activity, ARS staining, and the upregulation of key osteogenic markers. Similarly, in vivo experiments confirm FBLN1's capacity to promote bone regeneration in WJCMSCs, evidenced by increased CT bone volume, Masson staining results, and immunofluorescence staining for DSPP, DMP1, and OCN. Therefore, we want to explore the regulatory mechanism of FBLN1.

In the post‐transcriptional regulation of eukaryotic mRNA, 3′UTR exerts a pivotal role in the regulation of mRNA stability, degradation rate, and the efficiency of mRNA translation, which is involved in the RNA modifications or miRNA‐binding sties. It is well known that the 3′UTR region of mRNA contains the binding region of specific miRNAs. MiRNA, or microRNA, is indeed a class of endogenous small, single‐stranded noncoding RNA molecules (ncRNA), exerts its regulatory function by guiding the RNA‐induced silencing complex (RISC) to specifically target gene's 3′UTR in a complementary manner.^30^ Numerous studies have consistently demonstrated that miRNAs play a pivotal role in governing the process of osteogenic differentiation in MSCs.^31^, ^32^ In our research, we illustrated that miR615‐3p exerts a post‐transcriptional inhibitory effect on the expression of FBLN1 by forming complementary base pairs with the 3′UTR region of FBLN1 mRNA (Figure 4). It has been previously reported that miR615‐3p exerts a promotive effect on the progression of neonatal acute respiratory distress syndrome by suppressing the differentiation of MSCs into alveolar type epithelial cell.^33^ MiR615‐3p negatively regulates GDF5 and FOXO, thereby inhibiting the osteogenic differentiation of human lumbar ligamentum flavum cells.^34^ Moreover, miR615‐3p, which can regulate osteogenesis, is highly expressed in the microvesicles of human ADSCs.^29^ Our data demonstrates that miR615‐3p exerts negative regulation on the expression of FBLN1, leading to the inhibition of osteogenic differentiation in WJCMSCs. Moreover, the knockdown of miR615‐3p promoted the osteogenic differentiation of WJCMSCs by implantation experiments. These results suggested that miR615‐3p exerts regulatory effects on the osteogenic differentiation of WJCMSCs by targeting FBLN1 3′UTR and negatively control FBLN1. How miR615‐3p regulates 3′UTR of FBLN1 needs further study.

The m^6^A modification, as the most abundant and dynamic alteration in eukaryotic mRNA, exerts regulatory control over gene expression by modulating RNA splicing, translation efficiency, mRNA stability, nuclear‐cytoplasmic transport, and liquid–liquid phase separation.^25^, ^26^, ^35^ Our results revealed that the m^6^A modification in 3′UTR of FBLN1 mRNA exerts an inhibitory effect on the osteogenic differentiation of WJCMSCs by accelerating the mRNA degradation of FBLN1 (Figure 6). Recent research has revealed a negative correlation between the overall distribution of m^6^A peaks and miRNA‐binding sites in the 3′UTR of mRNA and m^6^A modification can affect the function of miRNAs in a certain spatial separation condition.^24^

mRNAs exhibiting enrichment in m^6^A modification demonstrate enhanced interaction with a greater number of miRNAs, whereby the target sites for these miRNAs are found in close proximity to the m6A sites within the mRNA molecules.^35^ Whether the co‐existence of m^6^A modification and miRNA‐binding sites in 3′UTR will affect each other's function remains unknown. In the present study, we show that mutation of the miR615‐3p‐binding site in FBLN1 mRNA affects its stability and the function of FBLN1 in promoting osteogenic differentiation of WJCMSCs independent on m^6^A site. Some reports suggest that m^6^A reader proteins impair the miRNA‐directed decay of target mRNAs by sequestering transcripts away from miRNA‐/RISC‐free conditions.^36^, ^37^, ^38^ However, our data reveal that miR615‐3p mediated the decay of FBLN1 mRNA dependent on the m^6^A reading function of YTHDF2, suggesting that recognizing the m^6^A site of FBLN1 is essential for miRNA‐mediated mRNA degradation.

Previous studies have shown that YTHDF2 plays a key role in osteogenesis and chondrogenesis through m^6^A modification recognition.^39^, ^40^ Studies have provided evidence indicating that YTHDF2 exerts a negative regulatory function in the differentiation of osteoclasts induced by LPS and also modulates the inflammatory response through the NF‐κB and MAPK signalling pathways.^39^ Moreover, the high expression of YTHDF2 in mouse haematopoietic stem cells promotes the attenuation of m^6^A‐modified Wnt mRNA, thereby inhibiting Wnt signalling function under the steady state and having a protective effect on inhibiting abnormal failure of haematopoietic stem cells.^40^ Here, we demonstrate that YTHDF2 promoted the attenuation of FBLN1 mRNA, thereby inhibiting the osteogenic differentiation of WJCMSCs. Mechanically, we found that YTHDF2 regulates FBLN1 protein expression by regulating FBLN1 mRNA degradation rather than inhibiting translation, which is consistent with the current mainstream research on the regulatory mechanism of YHDF2.^41^, ^42^ Recent studies have shown that YTHDF2 mediates two distinct degradation mechanisms for m^6^A‐containing mRNAs. One mechanism involves deadenylation mediated by the YTHDF2‐CCR4/NOT complex, while the other mechanism entails endoribonucleolytic cleavage facilitated by the YTHDF2‐HRSP12‐ribonuclease (RNase) P/mitochondrial RNA‐processing (MRP) complex.^43^ Our study failed to find evidence for direct binding of YTHDF2 to CNOT1 in WJCMSCs (data no‐show). Recent research has revealed that YTHDF1 facilitates mRNA degradation through its interaction with Ago2 and the process of phase separation.^44^ The miRNA‐loaded AGO is widely recognized as the essential component of the miRISC, which functions to facilitate translation repression and degradation of targeted mRNAs.^45^, ^46^ Nevertheless, it has been shown that m^6^A‐mediated miRNA‐mRNA recognition is independent of Ago proteins,^47^ suggesting the presence of additional regulatory proteins involved in facilitating the binding of a miRNA to its target mRNA under these circumstances. The results of the present study indicate that the m^6^A‐modification reading proteins YTHDF2 could form a complex with miR615‐3p and interact with FBLN1 3′UTR at the m^6^A site and the miRNA‐615‐3p‐binding site. Collectively, our findings indicate that YTHDF2 may be essential for the miR615‐3p‐mediated decay of FBLN1 mRNA in WJCMSCs. Moreover, our results showed that YTHDF2 could form a complex with AGO2 in WJCMSCs. Interestingly, the binding of YTHDF2 to AGO2 was attenuated when RNase A was added. Besides, miR615‐3p regulates FBLN1 3′UTR in a manner independent of binding to AGO proteins to form RISC. This suggests that there may be a novel mechanism for the YTHDF2‐mediated mRNA degradation. Here, we report a new mechanism of YTDHF2‐mediated RNA degradation involved of the interaction of m^6^A site and miRNA at the 3′UTR of target gene.

In summary, FBLN1 is critical in regulating the osteogenic differentiation potential and bone regeneration capacity of WJCMSCs. The regulation of FBLN1 depends on either the m^6^A site or the miR615‐3p‐binding site located in the 3′UTR of FBLN1 mRNA. Mechanistically, miR615‐3p is capable of interacting with both YTHDF2 and FBLN1 3′UTR, furthermore modulating the stability of FBLN1. This ultimately affect the osteogenic differentiation and bone regeneration ability of WJCMSCs. Our research has unveiled a novel m^6^A‐miRNA epigenetic regulatory pattern in MSCs for bone regeneration.

## AUTHOR CONTRIBUTIONS

Haoqing Yang, Wanqing Wang, Huina Liu and Chen Zhang: collection and assembly of data, data analysis and interpretation, manuscript writing; Yangyang Cao, Lujue Long, Xiao Han, and Yuejun Wang: collection and assembly of data, data analysis and interpretation; Fei Yan, Guoqing Li, and Mengyuan Zhu: assembly of data; Luyuan Jin and Zhipeng Fan: conception and design, final approval of manuscript, financial support. All authors have read and approved the final version of the manuscript.

## CONFLICT OF INTEREST STATEMENT

The authors declared no competing interests.

## Supporting information


**Data S1.** Supporting Information

## Data Availability

The data that support the findings of this study are available from the corresponding author upon reasonable request.
