# Supplementary material for: miR615‐3p inhibited FBLN1 and osteogenic differentiation of umbilical cord mesenchymal stem cells by associated with YTHDF2 in a m6A‐miRNA interaction manner
Source: Cell Prolif. 2024 Feb 14;57(6):e13607. doi: 10.1111/cpr.13607 (PMC11150146; doi:10.1111/cpr.13607)
Supplement: Supplementary file 1 — Data S1. Supporting Information [file CPR-57-e13607-s001.docx]

**Supplementary information**

**miR615-3p inhibited FBLN1 and** [**osteogenic differentiation**](javascript:;) **of umbilical cord mesenchymal stem cells by associated with** **YTHDF2 in a m6A-miRNA interaction manner**

**Supplementary Figures and figure legends**


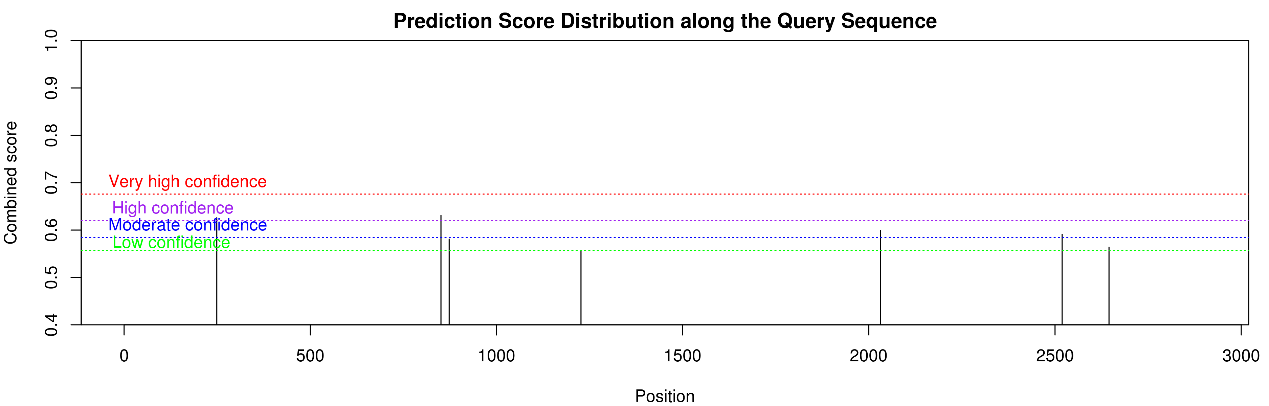


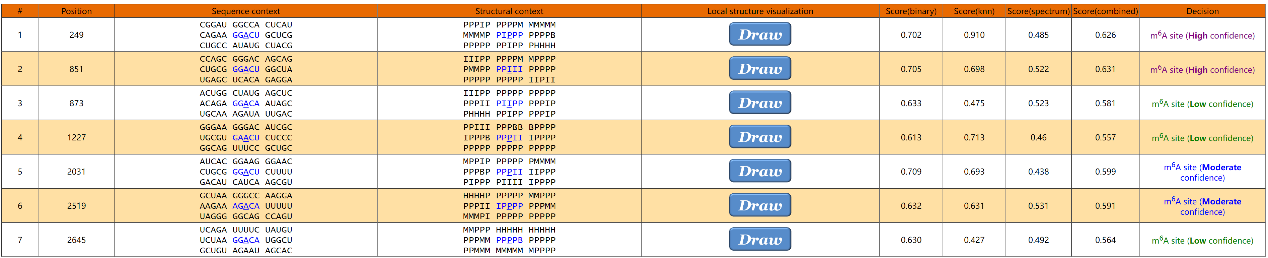


**Figure S1. SRAMP predicts m6A modification sites on FBLN1 mRNA.**


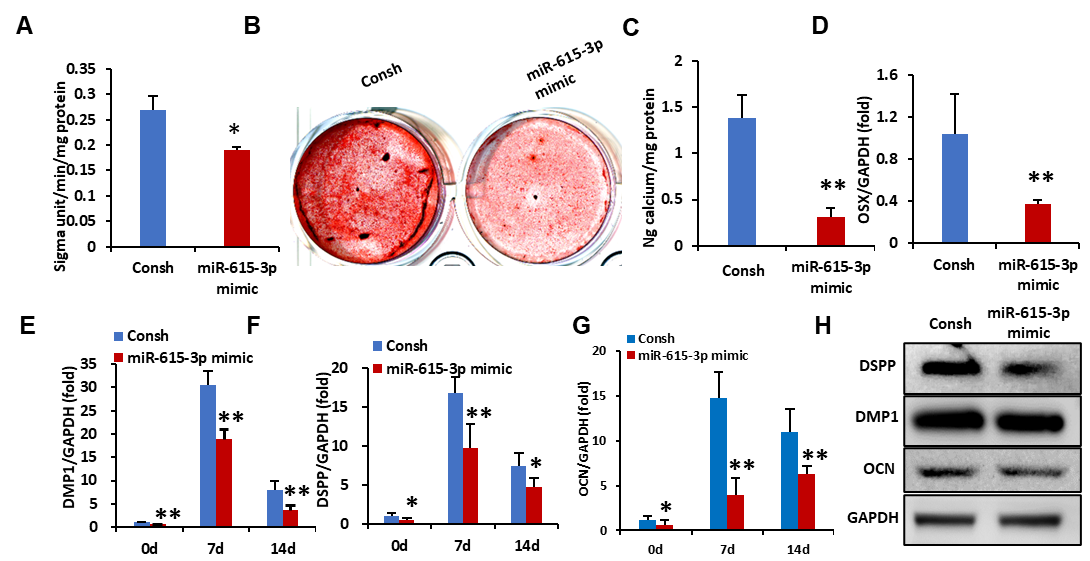


**Figure S2. miR-615-3p mimic negatively regulated the osteogenic differentiation of WJCMSCs.**  (A) miR-615-3p overexpression decreased ALP activity in WJCMSCs. (B, C) Alizarin red staining results. (D) qPCR results showed that overexpression of miR-615-3p downregulated the expression of OSX in WJCMSCs. (E-G) qPCR results showed that knockdown of miR-615-3p increased DMP1(E), DSPP(F) and OCN(G) on day 7 and day 14 in WJCMSCs after mineralization induction. (H) The protein expression levels of DSPP, DMP1 and OCN on day 14 after mineralization induction. GAPDH served as the internal control. Student’s t-test was performed to determine statistical significance. All error bars represent the s.d. (n=3). *P ≤ 0.05, **P ≤ 0.01.


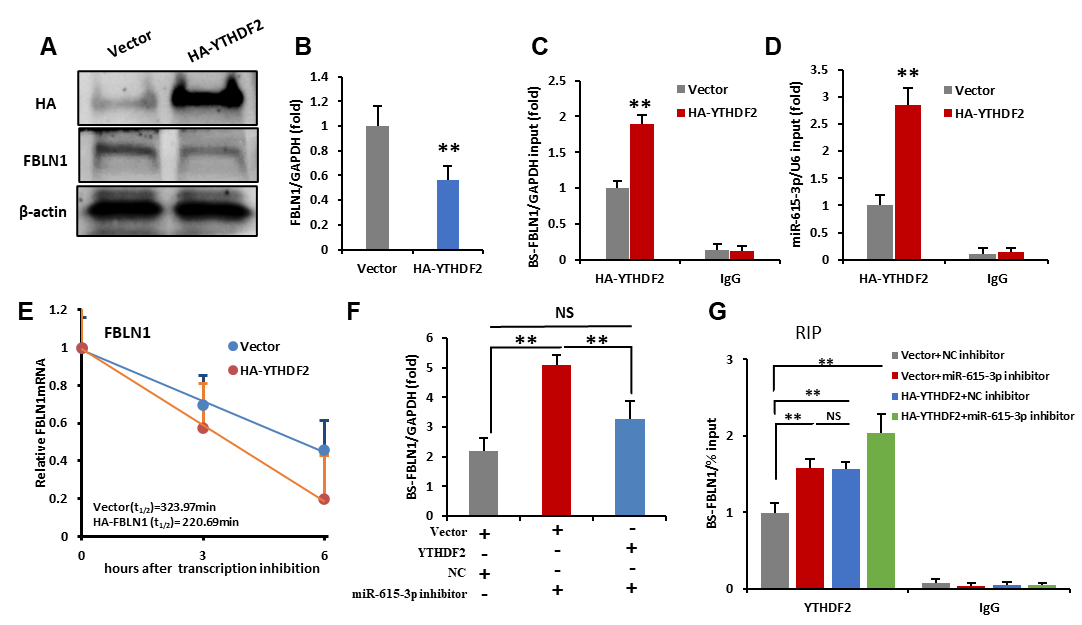


**Figure S3.** **YTHDF2 and miR-615-3p protein-RNA complex negatively regulates the stability of FBLN1.**

(A) The western blot results showed that YTHDF2 was over-expression and FBLN1 was downregulated in WJCMSCs/HA-YTHDF2 group. β-actin was used as a loading control. (B) qPCR results showed that FBLN1 was downregulated in WJCMSCs/HA-YTHDF2 group. (C) RIP results showed that FBLN1 mRNA could co-immunoprecipitate with more YTHDF2 protein in WJCMSCs/HA-YTHDF2 group. (D) RIP results showed that miR-615-3p could co-immunoprecipitate with more YTHDF2 protein in WJCMSCs/HA-YTHDF2 group. (E) Actinomycin D chase assay showed that the mRNA stability of FBLN1 in the WJCMSCs/Consh group was better than that in the WJCMSCs/HA-YTHDF2 group after Actinomycin D treatment (5 μg/mL) for 3 and 6 hours. (F) In WJCMSCs/HA-YTHDF2 group and WJCMSCs/Consh group, miR-615-3p inhibitors and control inhibitors were added to detect the expression of FBLN1 by qPCR, the results showed that the addition of mir615 inhibitors can promote the expression of FBLN1. (G) RIP results showed that FBLN1 mRNA could co-immunoprecipitate with YTHDF2 protein in WJCMSC/HA-YTHDF2 group, the addition of mir615 inhibitors can promote the binding state of YTHDF2 protein and FBLN1 mRNA. GAPDH was used as an internal control. Student’s t-test and One-way ANOVA were performed to determine statistical significance. All error bars represent the s.d. (n=3). *P ≤ 0.05. **P ≤ 0.01.


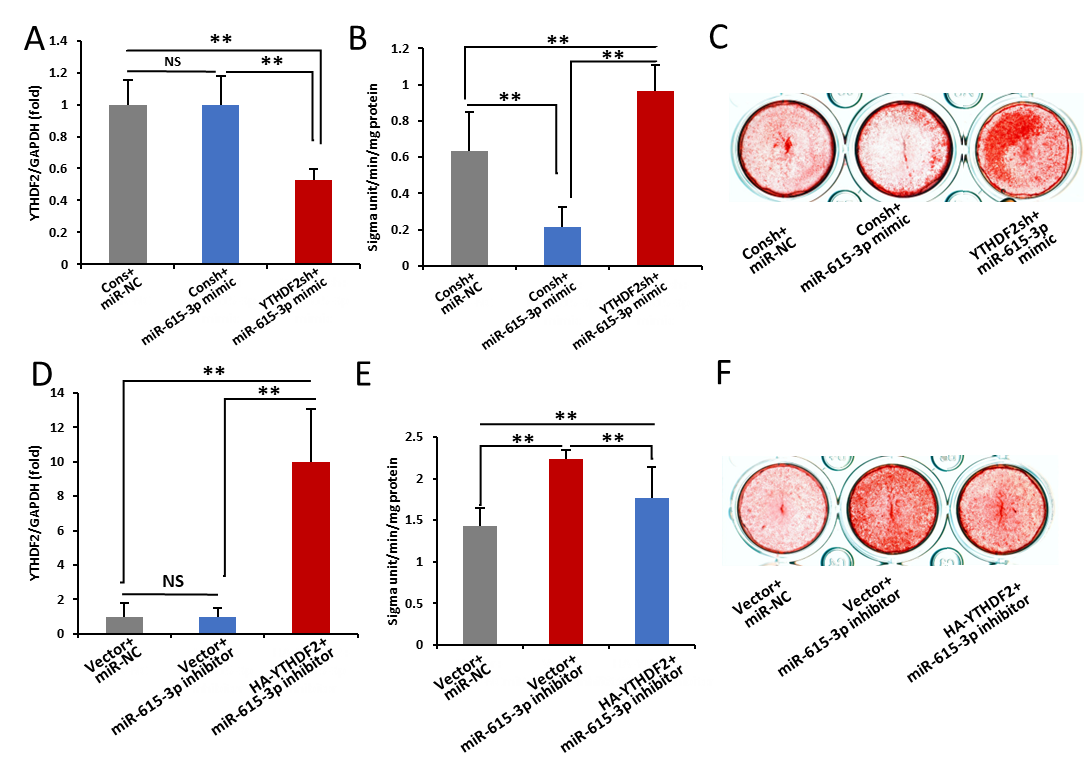


**Figure** **S4. YTHDF2 is essential for the osteogenic function of miR-615-3p.**

(A) In WJCMSCs/YTHDF2sh group and WJCMSCs/Consh group, miR-615-3p mimics and control mimics were added to detect the expression of YTHDF2 by qPCR, the results showed that the addition of miR-615-3p mimics did not change the expression of YTHDF2. (B) Even with the addition of miR-615-3p mimics, WJCMSCs/YTHDF2sh group showed higher ALP activity. (C) After the addition of miR-615-3p mimics, alizarin red staining showed that the mineralized nodule still increased in WJCMSCs/YTHDF2sh group. (D) In WJCMSCs/YTHDF2 group and WJCMSCs/Consh group, miR-615-3p inhibitors and control inhibitors were added to detect the expression of YTHDF2 by qPCR, the results showed that the addition of miR-615-3p inhibitors Suppressed the expression of YTHDF2. (E) With the addition of miR-615-3p inhibitors, WJCMSCs/YTHDF2 group showed lower ALP activity. (F) After the addition of miR-615-3p inhibitors, alizarin red staining showed that the mineralized nodule decreased in WJCMSCs/YTHDF2 group. GAPDH was used as an internal control. One-way ANOVA was performed to determine statistical significance. All error bars represent the s.d. (n=3). **P ≤ 0.01.


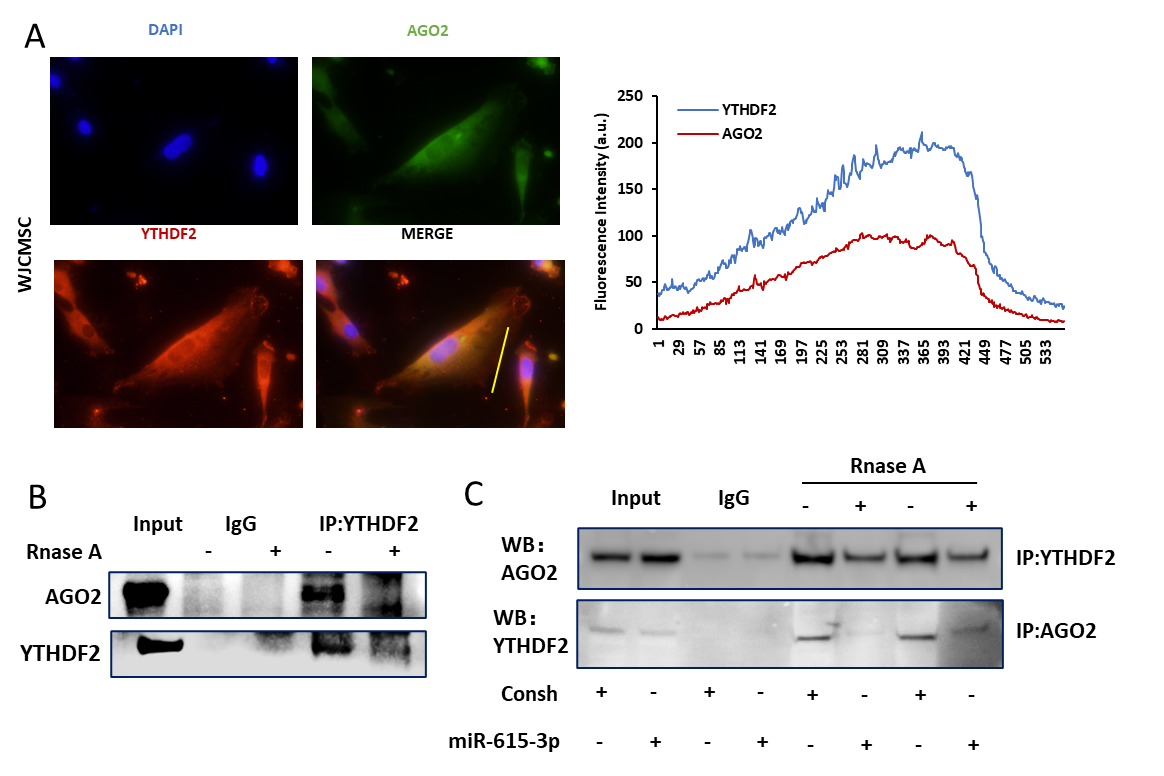


**Figure S5. YTHDF2 connects to AGO2 via miR-615-3p**

(A) Dual immunofluorescence staining and laser confocal detection of co-localization of YTHDF2 and AGO2 in WJCMSCs. The graph below showed the fluorescence intensity peaks along the line. (B) Co-immunoprecipitation (Co-IP) and western blot had shown the binding of YTHDF2 and AGO2 in Non-RNase A or RNase A treated WJCMSCs. (C) Co-immunoprecipitation (Co-IP) and western blot had shown the binding of YTHDF2 and AGO2 in Non-RNase A or RNase A treated WJCMSC/ miR-615-3p mimic group and WJCMSCs/Consh group.

| **Title** | **Description** |
| --- | --- |
| table S 1 | Primer sequences |
| table S 2 | RNA-Sequencing of microRNAs upon WJCMSCs |
| table S 3 | The MeRIP-seq results of the m6A modification sites on FBLN1 |
| table S 4 | The biotin-binding proteins were screened by biotin-miR615-3p in WJCMSCs |

| **SupplementaryTable1. Primers sequences used in the real-time RT-PCR** | |
| --- | --- |
| **Gene Symbol** | **Primer Sequences (5’—3”)** |
| GAPDH-F | CGGACCAATACGACCAAATCCG |
| GAPDH-R | AGCCACATCGCTCAGACACC |
| FBLN1-F | TGCCATGAGAATCGGGAGTG |
| FBLN1-R | GGTGGTGAAAAAGCCCTCCT |
| OSX-F | CCTCCTCAGCTCACCTTCTC |
| OSX-R | GTTGGGAGCCCAAATAGAAA |
| DSPP-F | CGACATAGGTCACAATGAGGATGTCG |
| DSPP-R | TTGCTTCCAGCTACTTGAGGTC |
| DMP1-F | CGTGGACAAAGAAGATAGCAACTCCACG |
| DMP1-R | TTCCGGCTCTCTATCTCAATGTTT |
| FBLN1-BS-F | GCACCCAGTTCATGATAGGC |
| FBLN1-BS-R | CCAGGCTGTTGTGCAAGTT |
| YTHDF2-F | GTTGGTAGCGGGTCCATTACT |
| YTHDF2-R | GGTCTTCAGTTTAGGTTGCTGT |
| FBLN1-CLIP1-F | CATGTATTAAGCTGAGCCAGATGA |
| FBLN1-CLIP1-R | ACACCGAAAATACATCAGATGGAC |
| FBLN1-CLIP2-F | GAAGGCTAAGTGTCACCCCC |
| FBLN1-CLIP2-R | TCTTTCCTTGGCCCTTAGCA |
| FBLN1-CLIP3-F | CCTTGCTAAGGGCCAAGGAA |
| FBLN1-CLIP3-R | GACTGGCTGCCCCCTAAAAA |
| FBLN1-CLIP4-F | TTTTTAGGGGGCAGCCAGTC |
| FBLN1-CLIP4-R | TCAGGGCAAGAACTGGTCTTC |
| FBLN1-CLIP5-F | TGTTCTAAGGACATGGCTGCT |
| FBLN1-CLIP5-R | ATTTATCATCCACGTCTGTGCT |
| FBLN1-CLIP6-F | ATCCCCAGAAGCAGCATGAC |
| FBLN1-CLIP6-R | TTTCCCTTCCAAGTGCTCCC |
| FBLN1-CLIP7-F | GGGAGCACTTGGAAGGGAAA |
| FBLN1-CLIP7-R | AACATACTGGAGGCCCACAC |
| FBLN1-CLIP8-F | GGGCCTCCAGTATGTTCACC |
| FBLN1-CLIP8-R | GATGATGCAGAGTGCCGAGA |

**Table S2: RNA-Sequencing of microRNAs upon WJCMSCs**

| Probe Set ID | P.value | FC | Regulation | Species Scientific Name | Sequence Type | Sequence Source | Transcript ID(Array Design) |
| --- | --- | --- | --- | --- | --- | --- | --- |
| 20500418 | 0.722185 | 0.768772 | down | Homo sapiens | miRNA | miRBase | hsa-miR-129-5p |
| 20500743 | 0.48062 | 0.886471 | down | Homo sapiens | miRNA | miRBase | hsa-miR-138-5p |
| 20503105 | 0.162983 | 0.335864 | down | Homo sapiens | miRNA | miRBase | hsa-miR-486-5p |
| 20503106 | 0.247577 | 0.200028 | down | Homo sapiens | miRNA | miRBase | hsa-miR-486-3p |
| 20503809 | 0.865767 | 1.247849 | up | Homo sapiens | miRNA | miRBase | hsa-miR-497-5p |
| 20517915 | 0.276499 | 0.557394 | down | Homo sapiens | miRNA | miRBase | hsa-miR-3663-5p |
| 20519079 | 0.156619 | 0.740777 | down | Homo sapiens | miRNA | miRBase | hsa-miR-3977 |
| 20519521 | 0.613569 | 0.935573 | down | Homo sapiens | miRNA | miRBase | hsa-miR-4704-5p |
| 20520348 | 0.387918 | 0.829631 | down | Homo sapiens | miRNA | miRBase | hsa-miR-5095 |
| 20536471 | 0.263794 | 0.77544 | down | Homo sapiens | stem-loop | miRBase | hsa-mir-4253 |
| 20500136 | 0.429188 | 1.456633 | up | Homo sapiens | miRNA | miRBase | hsa-miR-19b-1-5p |
| 20500393 | 0.000702 | 127.3487 | up | Homo sapiens | miRNA | miRBase | hsa-miR-196a-5p |
| 20500440 | 0.008202 | 94.31599 | up | Homo sapiens | miRNA | miRBase | hsa-miR-10b-5p |
| 20500464 | 0.839932 | 0.721991 | down | Homo sapiens | miRNA | miRBase | hsa-miR-210-5p |
| 20500742 | 0.130882 | 6.556225 | up | Homo sapiens | miRNA | miRBase | hsa-miR-137 |
| 20500790 | 0.285684 | 1.380523 | up | Homo sapiens | miRNA | miRBase | hsa-miR-186-3p |
| 20501036 | 0.848405 | 1.606698 | up | Homo sapiens | miRNA | miRBase | hsa-miR-200c-3p |
| 20501242 | 0.009817 | 4.587123 | up | Homo sapiens | miRNA | miRBase | hsa-miR-378a-5p |
| 20501771 | 0.027491 | 24.19478 | up | Homo sapiens | miRNA | miRBase | hsa-miR-196b-5p |
| 20501772 | 0.159439 | 1.713887 | up | Homo sapiens | miRNA | miRBase | hsa-miR-196b-3p |
| 20502451 | 0.203626 | 2.424564 | up | Homo sapiens | miRNA | miRBase | hsa-miR-452-5p |
| 20503801 | 0.150974 | 1.496477 | up | Homo sapiens | miRNA | miRBase | hsa-miR-432-3p |
| 20503847 | 0.392434 | 4.925147 | up | Homo sapiens | miRNA | miRBase | hsa-miR-517a-3p |
| 20503853 | 0.392434 | 4.925147 | up | Homo sapiens | miRNA | miRBase | hsa-miR-517b-3p |
| 20504357 | 0.001831 | 83.71581 | up | Homo sapiens | miRNA | miRBase | hsa-miR-615-3p |
| 20504557 | 0.216014 | 1.227752 | up | Homo sapiens | miRNA | miRBase | hsa-miR-767-5p |
| 20506004 | 0.282761 | 24.36016 | up | Homo sapiens | miRNA | miRBase | hsa-miR-935 |
| 20506716 | 0.651148 | 1.64752 | up | Homo sapiens | miRNA | miRBase | hsa-miR-1184 |
| 20506821 | 0.727058 | 1.973011 | up | Homo sapiens | miRNA | miRBase | hsa-miR-1293 |
| 20517677 | 0.684376 | 1.098894 | up | Homo sapiens | miRNA | miRBase | hsa-miR-4294 |
| 20535307 | 0.143597 | 1.292419 | up | Homo sapiens | stem-loop | miRBase | hsa-mir-551b |
| 20536952 | 0.473063 | 1.213173 | up | Homo sapiens | stem-loop | miRBase | hsa-mir-4718 |

**Table S3: The MeRIP-seq results of the m6A modification sites on FBLN1**

| ensembl_gene_id | chr | chromStart | chromEnd | score | strand | thickStart | thickEnd | itemRgb | blockCount | blockSizes | blockStarts | lg.p | lg.fdr | fold_enrchment | class | external_gene_name | gene_biotype | SingeSitemotif: GGAC |
| --- | --- | --- | --- | --- | --- | --- | --- | --- | --- | --- | --- | --- | --- | --- | --- | --- | --- | --- |
| ENSG00000077942 | 22 | 45502970 | 45502970 | 4.40E-09 | + | 45502880 | 45503061 | 0 | 1 | 181, | 0 | -8.36 | -7.62 | 2.86 | five_prime_utr | FBLN1 | protein_coding | 45502933, |
| ENSG00000077942 | 22 | 45600849 | 45600849 | 4.90E-07 | + | 45600744 | 45600955 | 0 | 1 | 211, | 0 | -6.31 | -5.61 | 2.29 | three_prime_utr | FBLN1 | protein_coding | 45600875, |
| ENSG00000077942 | 22 | 45503000 | 45503000 | 0.00091 | + | 45502910 | 45503091 | 0 | 1 | 181, | 0 | -3.04 | -2.54 | 11 | CDS | FBLN1 | protein_coding | 45502933, |

**Table S4: The biotin-binding proteins were screened by biotin-miR615-3p in WJCMSCs**

| N | Unused | Total | %Cov | %Cov(50) | %Cov(95) | Accession | Name | Species | Peptides(95%) |
| --- | --- | --- | --- | --- | --- | --- | --- | --- | --- |
| 16 | 0 | 10.28 | 16.08 | 10.65 | 10.65 | tr\|H0YDD4\|H0YDD4_HUMAN | Acetyltransferase component of pyruvate dehydrogenase complex (Fragment) OS=Homo sapiens GN=DLAT PE=1 SV=1 | HUMAN | 8 |
| 35 | 7.96 | 8.12 | 14.92 | 13.72 | 12.69 | tr\|A0A087X0Q1\|A0A087X0Q1_HUMAN | YTH domain-containing family protein 3 OS=Homo sapiens GN=YTHDF3 PE=1 SV=1 | HUMAN | 7 |
| 15 | 0 | 9.83 | 28.44 | 21.25 | 14.37 | tr\|Q5VVL7\|Q5VVL7_HUMAN | Lipoamide acyltransferase component of branched-chain alpha-keto acid dehydrogenase complex, mitochondrial OS=Homo sapiens GN=DBT PE=1 SV=1 | HUMAN | 6 |
| 33 | 0 | 8.51 | 17.06 | 13.55 | 13.55 | sp\|P08559-4\|ODPA_HUMAN | Isoform 4 of Pyruvate dehydrogenase E1 component subunit alpha, somatic form, mitochondrial OS=Homo sapiens GN=PDHA1 | HUMAN | 6 |
| 80 | 2.91 | 4.97 | 10.73 | 7.642 | 7.642 | tr\|B1ANR0\|B1ANR0_HUMAN | Poly(A) binding protein, cytoplasmic 4 (Inducible form), isoform CRA_e OS=Homo sapiens GN=PABPC4 PE=1 SV=1 | HUMAN | 5 |
| 34 | 0 | 6.34 | 29.63 | 29.63 | 29.63 | tr\|E7ETC0\|E7ETC0_HUMAN | Nucleolysin TIAR OS=Homo sapiens GN=TIAL1 PE=1 SV=1 | HUMAN | 5 |
| 30 | 9.24 | 9.29 | 5.793 | 5.793 | 4.786 | tr\|D6REM6\|D6REM6_HUMAN | Matrin-3 OS=Homo sapiens GN=MATR3 PE=1 SV=1 | HUMAN | 5 |
| 122 | 1.75 | 4.69 | 11.95 | 10.72 | 6.854 | tr\|A0A087WXZ3\|A0A087WXZ3_HUMAN | YTH domain-containing family protein 2 OS=Homo sapiens GN=YTHDF2 PE=1 SV=1 | HUMAN | 4 |
| 40 | 6.91 | 7.01 | 10.33 | 9.335 | 5.941 | sp\|P23246\|SFPQ_HUMAN | Splicing factor, proline- and glutamine-rich OS=Homo sapiens GN=SFPQ PE=1 SV=2 | HUMAN | 4 |
| 36 | 7.64 | 7.64 | 21.64 | 21.64 | 21.64 | tr\|A0A0A0MSI0\|A0A0A0MSI0_HUMAN | Peroxiredoxin-1 (Fragment) OS=Homo sapiens GN=PRDX1 PE=4 SV=1 | HUMAN | 4 |
| 51 | 0 | 5.13 | 8.143 | 6.84 | 6.84 | sp\|P02545-6\|LMNA_HUMAN | Isoform 6 of Prelamin-A/C OS=Homo sapiens GN=LMNA | HUMAN | 4 |
| 43 | 6.69 | 6.83 | 12.13 | 7.807 | 7.807 | sp\|Q07065\|CKAP4_HUMAN | Cytoskeleton-associated protein 4 OS=Homo sapiens GN=CKAP4 PE=1 SV=2 | HUMAN | 4 |
| 47 | 6.03 | 6.03 | 8.958 | 5.212 | 5.212 | tr\|J3KTA4\|J3KTA4_HUMAN | Probable ATP-dependent RNA helicase DDX5 OS=Homo sapiens GN=DDX5 PE=1 SV=1 | HUMAN | 3 |
| 83 | 2.74 | 2.84 | 5.503 | 5.503 | 3.605 | tr\|A6NLN1\|A6NLN1_HUMAN | Polypyrimidine tract binding protein 1, isoform CRA_b OS=Homo sapiens GN=PTBP1 PE=1 SV=4 | HUMAN | 3 |
| 48 | 0 | 3.99 | 10.61 | 6.552 | 4.524 | tr\|M0R3F1\|M0R3F1_HUMAN | Heterogeneous nuclear ribonucleoprotein U-like protein 1 (Fragment) OS=Homo sapiens GN=HNRNPUL1 PE=1 SV=1 | HUMAN | 3 |
| 98 | 2.15 | 2.17 | 8.889 | 8.889 | 8.444 | tr\|M0R0R2\|M0R0R2_HUMAN | 40S ribosomal protein S5 OS=Homo sapiens GN=RPS5 PE=1 SV=1 | HUMAN | 3 |
| 52 | 2.08 | 2.09 | 6.736 | 6.736 | 5.181 | tr\|F6Y5H0\|F6Y5H0_HUMAN | RNA-binding motif, single-stranded-interacting protein 1 OS=Homo sapiens GN=RBMS1 PE=1 SV=1 | HUMAN | 2 |
| 90 | 0 | 1.15 | 12.5 | 12.5 | 12.5 | tr\|C9J5V9\|C9J5V9_HUMAN | Nuclease-sensitive element-binding protein 1 (Fragment) OS=Homo sapiens GN=YBX1 PE=1 SV=1 | HUMAN | 2 |
| 121 | 1.8 | 3.32 | 8.213 | 8.213 | 8.213 | tr\|C9IZL7\|C9IZL7_HUMAN | Non-POU domain-containing octamer-binding protein (Fragment) OS=Homo sapiens GN=NONO PE=1 SV=1 | HUMAN | 2 |
| 95 | 2.21 | 2.24 | 11.93 | 11.93 | 11.93 | sp\|P31025\|LCN1_HUMAN | Lipocalin-1 OS=Homo sapiens GN=LCN1 PE=1 SV=1 | HUMAN | 2 |
| 77 | 0 | 3.32 | 7.331 | 5.279 | 5.279 | sp\|P22626-2\|ROA2_HUMAN | Isoform A2 of Heterogeneous nuclear ribonucleoproteins A2/B1 OS=Homo sapiens GN=HNRNPA2B1 | HUMAN | 2 |
| 92 | 0 | 2.41 | 3.473 | 1.23 | 1.23 | sp\|P35579-2\|MYH9_HUMAN | Isoform 2 of Myosin-9 OS=Homo sapiens GN=MYH9 | HUMAN | 2 |
| 75 | 0 | 3.43 | 7.303 | 5.337 | 5.337 | sp\|P51991-2\|ROA3_HUMAN | Isoform 2 of Heterogeneous nuclear ribonucleoprotein A3 OS=Homo sapiens GN=HNRNPA3 | HUMAN | 2 |
| 66 | 0 | 4.04 | 7.649 | 5.666 | 5.666 | sp\|Q15717-2\|ELAV1_HUMAN | Isoform 2 of ELAV-like protein 1 OS=Homo sapiens GN=ELAVL1 | HUMAN | 2 |
| 78 | 3.12 | 3.24 | 5.95 | 4.793 | 3.306 | tr\|F8W930\|F8W930_HUMAN | Insulin-like growth factor 2 mRNA-binding protein 2 OS=Homo sapiens GN=IGF2BP2 PE=1 SV=1 | HUMAN | 2 |
| 87 | 2.51 | 2.59 | 5.172 | 3.664 | 3.664 | tr\|B7Z645\|B7Z645_HUMAN | Heterogeneous nuclear ribonucleoprotein Q OS=Homo sapiens GN=SYNCRIP PE=1 SV=1 | HUMAN | 2 |
| 70 | 3.71 | 3.78 | 6.776 | 4.673 | 4.673 | tr\|Q5T6W5\|Q5T6W5_HUMAN | Heterogeneous nuclear ribonucleoprotein K OS=Homo sapiens GN=HNRNPK PE=1 SV=1 | HUMAN | 2 |
| 144 | 1.29 | 3.6 | 7.229 | 7.229 | 5.301 | sp\|P52597\|HNRPF_HUMAN | Heterogeneous nuclear ribonucleoprotein F OS=Homo sapiens GN=HNRNPF PE=1 SV=3 | HUMAN | 2 |
| 53 | 4.92 | 5 | 9.379 | 5.763 | 1.921 | sp\|Q8IYB7\|DI3L2_HUMAN | DIS3-like exonuclease 2 OS=Homo sapiens GN=DIS3L2 PE=1 SV=4 | HUMAN | 2 |
| 101 | 0 | 1.68 | 3.822 | 3.822 | 3.822 | tr\|G3V153\|G3V153_HUMAN | Caprin-1 OS=Homo sapiens GN=CAPRIN1 PE=1 SV=1 | HUMAN | 2 |
| 145 | 1.29 | 1.49 | 6.221 | 3.226 | 3.226 | sp\|P06733\|ENOA_HUMAN | Alpha-enolase OS=Homo sapiens GN=ENO1 PE=1 SV=2 | HUMAN | 2 |
